# Salvianolic acid B activates chondrocytes autophagy and reduces chondrocyte apoptosis in obese mice via the KCNQ1OT1/miR-128-3p/SIRT1 signaling pathways

**DOI:** 10.1186/s12986-022-00686-0

**Published:** 2022-08-03

**Authors:** Tianwen Sun, Fei Wang, Gaojian Hu, Zhizhou Li

**Affiliations:** Department of Orthopedics, China-Japan Union Hospital of Jilin University, No. 126 of Xiantai Street, Changchun, 130021 Jilin Province China

**Keywords:** Salvianolic acid B, KCNQ1OT1, Obesity, Autophagy, Osteoarthritis

## Abstract

**Purpose:**

Salvianolic acid B (Sal B) possesses strong anti-inflammatory and antioxidant activity. This study aims to explore the underlying mechanism of Sal B to improve the obesity-related osteoarthritis (OA).

**Methods:**

C57BL/6 J male mice were fed with a normal control diet (NCD), a high fat diet (HFD), or HFD with Sal B (25 mg/kg), and mouse body weights and osteoarticular inflammatory factor levels were examined. Mouse chondrogenic cell line ATDC5 were transfected with lncRNA KCNQ1 overlapping transcript 1 small hairpin RNA (KCNQ1OT1 shRNA), miR-128-3p mimic or Sirtuin-1 small interfering RNA (SIRT1 siRNA), then stimulated with Palmitic acid (PA) followed by the treatment of Sal B. Then, inflammatory response, apoptosis, and autophagy of ATDC5 cells in different groups were detected.

**Results:**

Sal B reduced the body weight, decreased the levels of inflammatory markers, and improved cartilage damage in OA mice fed with HFD. KCNQ1OT1 was downregulated in OA mice fed with HFD, and PA-stimulated ATDC5 cells. Sal B protected ATDC5 cells against PA-mediated inflammation, apoptosis, and the inhibition of autophagy, while knockdown of KCNQ1OT1 reversed these results. KCNQ1OT1 was found to be functioned as a ceRNA to bind and downregulate the expression of miR-128-3p that was upregulated in PA-induced cells. Furthermore, SIRT1 was verified as a target of miR-128-3p. MiR-128-3p overexpression reversed the effects of Sal B on inflammatory response, apoptosis, and autophagy in PA-stimulated cells, and knockdown of SIRT1 displayed the similar results.

**Conclusion:**

Sal B exerted a chondroprotective effect by upregulating KCNQ1OT1, which indicates Sal B can used for a therapeutic agent in obesity-related OA.

**Supplementary Information:**

The online version contains supplementary material available at 10.1186/s12986-022-00686-0.

## Introduction

Osteoarthritis (OA) is a chronic joint disease that is characterized by cartilage damage, subchondral bone sclerosis, and synovial tissue inflammation [1]. Obesity, which leads to joint overloading and chronic low-grade inflammation, is considered to be a major risk factor of OA occurrence [42]. In recent years, the prevalence of osteoarthritis is evidently increased with the rising obesity. Currently, drug therapy and non-drug therapy have been used in OA treatment to improve symptoms, reduce pain, prevent joint degeneration, and maintain joint function. However, long-term use of drugs can result in the occurrence of side effects that are related to gastrointestinal, kidney and cardiovascular diseases [12]. Therefore, there is an urgent need to develop new therapeutic drugs and their targets that can slow and/or reverse cartilage degradation [7].

Primary (spontaneous) and secondary (post-traumatic OA, including induced models) OA have been used as OA models [20]. Anterior cruciate ligament (ACL) tears are commonly correlated with the impairment of the articular cartilage, menisci, subchondral bone, and other ligaments, which induces posttraumatic OA based on its ability destabilize knee joints [16]. Moreover, total or partial meniscectomy disturbs the natural loading mechanism of the knee joint, which increases the strain on articular cartilage and mimics OA development. It is reported that a high fat/high sucrose diet is an independent risk factor for OA in rats with anterior cruciate ligament-transected (ACL-T) knees [9].

Salvianolic acid B (Sal B) is a water-soluble polyphenolic acid extracted from the traditional Chinese medicine herb *Salvia miltiorrhiza*. Sal B exhibits multiple bioactivities, including the reduction of inflammatory response, inhibition of apoptosis and alleviation of oxidative stress [23, 24], thus treating a great range of diseases in traditional and modern medicine, such as breast cancer, diabetes, and atherosclerosis [18, 19, 39]. Sal B may reduce the infiltration of neutrophils after sepsis caused by CLP, and improve the pathological changes and inflammation of the liver induced by sepsis [30]. Yang et al. reports that Sal B could inhibit the inflammatory response in endothelial cells and pericytes via the YAP/TAZ/JNK signaling pathway to improve atherosclerosis [39]. Importantly, Sal B is reported to suppress IL-1β-induced inflammatory reactions in human OA chondrocytes via the inhibition of NF-κB signaling, thus reducing cartilage degradation [27]. Moreover, recent studies showed that acid B can reduce obesity and obesity-related metabolic disorders [28, 35, 46]. However, there is insufficient information regarding the protective functions of Sal B in obesity-related OA.

Long noncoding RNAs (lncRNAs) are a class of functional RNA molecules (≥ 200 nts) that can function to regulate gene expression through epigenetic modification, transcriptional regulation, and post-transcriptional regulation [32]. Increasing evidence indicates that lncRNAs act as competing endogenous RNAs (ceRNAs) to sponge miRNAs to participate the development of OA. For example, lncRNA PART1 regulates chondrocyte proliferation, apoptosis, and extracellular matrix degradation in osteoarthritis via miR-373-3p/SOX4 axis [48]. LncRNA NKILA could promote proliferation and suppress apoptosis of chondrocytes via miR-145/SP1/NF-κB signaling in human osteoarthritis [37]. Furthermore, lncRNA KCNQ1 overlapping transcript 1 (KCNQ1OT1) is reported to be involved in regulating bone metabolism. It is reported that KCNQ1OT1 enhance osteoblasts proliferation and migration and inhibit apoptosis through the regulation of the miR-701-3p/FGFR3 axis [8]. Yu et al. verified that knockdown of KCNQ1OT1 protects TSCs (Tendon stem cells) from adipogenic and osteogenic differentiation through increasing the expression of miR-138 and downregulating the expressions of PPARγ and RUNX2 [41]. Moreover, Sal B is verified to regulate the expression of lncRNAs associated with adipocyte differentiation, lipid metabolism, and inflammation, which suggesting that Sal B exerts an important role in inhibiting obesity by regulating anti-inflammatory related factors and signaling pathways [4]. Thus, we reasonably speculate that there is a link between KCNQ1OT1 and Sal B-mediated protective effect in obesity-related OA.

The aim of this study is to explore the role of Sal B in obesity-related OA and the possible molecular mechanism, which may provide a new therapeutic target for its application and development.

## Materials and methods

### Sample collection

The degenerated cartilage tissues were obtained from the knee joints of 40 patients (age, 57.54 ± 8.9 years) who underwent total knee arthroplasty. The normal cartilage tissues were collected from 40 volunteers (age, 38.94 ± 4.2 years) with the femoral neck fracture with no history of rheumatoid arthritis or OA. The body mass index (BMI) of all selected patients is 28.5 ± 3.1 kg/m^2^ which meets the obesity diagnostic criteria of greater than or equal to 23 kg/m^2^. These cartilage samples were immediately snap-frozen and stored in the liquid nitrogen for further experiments. The present study was approved by the Clinical Research Ethics of China-Japan Union Hospital of Jilin University.

### Animals and experimental group

Seven-week-old C57BL/6 male wild-type (WT) mice were purchased from the Animal Center of the Chinese Academy of Sciences (Shanghai, China). After one week of acclimatization, the mice were randomly divided into four groups of 12 mice. Three test groups were fed with a diet composed of 60.0% kcal from fat, 20.0% kcal from carbohydrates, and 20.0% kcal from protein to constitute the high-fat diet (HFD) groups, and the remaining group was fed with a composed of 10.0% kcal from fat, 70.0% kcal from carbohydrates, and 20.0% kcal from protein to constitute the normal control diet (NCD) group from week 1 to week 21. The HFD and NCD used in this study were provided by HFK Bioscience Co., LTD (Beijing, China). After the HFD induction for 12 weeks, the mice were randomly divided into three groups: HFD + Sham group (surgery with an opened knee-joint capsule without ACLT + MMx) and two OA groups (HFD + OA + Vechicle and HFD + OA + Sal B; surgery with ACLT + MMx). One day after the operation, the mice in HFD + OA + Sal B group were received an intraperitoneal-injection of Sal B (25 mg/kg) daily for 10 weeks, whereas the mice in HFD + OA + Vechicle group were injected with DMSO. Mice were housed in chambers with natural light at controlled temperature of 24 ± 1 °C and 40–60% humidity. Body weights were measured once a week for a total of 21 weeks, and the widths of the knee joints were measured weekly by calipers from 13 to 21 week. Mice were euthanized with CO_2_ at the end of 20 week. Immediately, blood samples were collected and centrifuged for 30 min at 10,000 × *g* at 4 °C. Then, the obtained supernatant was stored at − 80 °C for further experiments. And the knees of mice were dissected after all tests were completed.

All of the experimental procedures involving animal care and use met the Guidelines set forth by the Chinese National Institutes of Health, and was approved by the local Institutional Animal Care Ethics Committee for animal studies at China-Japan Union Hospital of Jilin University.

### Osteoarthritis animal model

The surgery with ACLT/MMx were performed to establish the rat model of OA as described by period studies [31]. After anesthetization with 3% pentobarbital sodium (Tocris, Avonmouth, UK), the hair on the right knee was clipped. Right knee was subsequently exposed before an incision was made in the medial aspect of the joint capsule (anterior to the medial collateral ligament), then the anterior cruciate ligament was transected, and the medial meniscus was completely resected in a manner that did not injure the articular cartilage. Subsequently, the joint was irrigated with normal saline, the capsule was sutured with 4–0 chromic catgut, and the skin was closed with 4–0 nylon mattress sutures. And the mice were allowed to move, eat and drink freely after surgery. The control group (Sham group) received sham operations involving an arthrotomy but without transecting anterior cruciate ligament and removing medial meniscus.

### Histological analysis

The collected knee joints were fixed in brown vials with 4% paraformaldehyde for 2 days, then decalcified with 10% ethylenediaminetetraacetic acid (EDTA, PH 7.2) for 4 weeks. After decalcification, the joints were embedded in paraffin blocks and sagittally cut into sections at a thickness of 5 mm. The sections were then dewaxed in xylene and hydrated with graded ethanol series. Hematoxylin/eosin (H&E) staining (Tianjin Guangfu Fine Chemical Research Institute, Tianjin, China) was then used to examine the morphological changes. The histological examinations of cartilage were blindly evaluated according to the grading of Osteoarthritis Research Society International (OARSI) scoring system [11]. The cartilage matrix loss width, the cartilage degeneration score, the total and significant cartilage degeneration widths, and the zonal depth ratio of the lesions were specifically evaluated.

### Cell culture and treatments

The mouse chondrocyte cell line ATDC5 was purchased from Shanghai Institute of Biosciences Cell Resource Center, Chinese Academy of Sciences. Cells were cultured in in Dulbecco Modified Eagle Medium/Ham Nutrient Mixture F12 (DMEM/F12; Gibco, Life Technologies, Carlsbad, CA) containing 10% fetal bovine serum (FBS, Invitrogen, Carlsbad, CA, USA) and 1% penicillin/streptomycin (Gibco, Life Technologies). The cells were maintained in a humidified incubator with 5% CO_2_ at 37 °C. All these ATDC5 cells were used between the fifth and tenth passages.

Palmitic acid (PA) was purchased from Sigma-Aldrich. PA with a series of concentrations (100 µM, 200 µM, 300 µM, 400 µM and 500 µM) were used to treat ATDC5 cells for 24 h to stimulate inflammatory injury. Sal B (purity N98%) was purchased from Shanghai Winherb Medical Science Co., Ltd. (Shanghai, China), and dissolved in DMSO (≥ 99.7%, Sigma-Aldrich). ATDC5 cells were treated in the absence or presence of 25/50/or 100 μM Sal B for 24 h after PA treatment. DMSO (≥ 99.7%, Sigma-Aldrich) without Sal B was used as control group.

### Cell transfection

The gene-overexpression vector (Ad-KCNQ1OT1) and the control vector (Vector) were purchased from GenScript Biotech Corp. (Nanjing, China). The small interfering RNAs against SIRT1 (SIRT1 siRNA) and negative control siRNAs (NC siRNA) were designed, synthesized and validated by Thermo Fisher Scientific (Waltham, MA, USA). MiR-128a-3p mimic, miR-128-3p inhibitor and the corresponding negative control (NC mimic and NC inhibitor) were designed and synthesized by GenePharma Corporation (Shanghai, China). All these plasmids and oligonucleotides were transfected into ATDC5 chondrocyte cells by using lipofectamine 2000 transfection reagent (Invitrogen, Carlsbad, USA) following the guidelines of the manufacturer. At 48 h after transfection, cells were harvested for further study.

### RNA extraction and quantitative real-time polymerase chain reaction (qRT-PCR)

Total RNAs were extracted from treated ATDC5 cells and cartilage tissue using RNA pure Total RNA Fast Extraction Kit (Sangon, Shanghai, China), and reversely transcribed to cDNA by PrimeScript RT reagent Kit (Thermo Fisher Scientific, Waltham, MA, USA). The quantitative analysis of KCNQ1OT1 and SIRT1 were performed by the SYBRTM Green PCR Master Mix (Applied Biosystems, Foster City, CA, USA) with β -actin as an endogenous control. The levels of miR-128-3p were analyzed by SYBR PrimeScript miRNA RT‐PCR Kit (Takara Biotechnology, Dalian, China) with U6 as the internal reference. qRT-PCR was conducted on CFX96 qPCR machine (Invitrogen, Carlsbad, CA, USA) with the following steps: 10 min at 95 °C; 35 cycles of 15 s at 95 °C, 20 s at 60 °C and 15 s at 72 °C. Data were quantified using 2^−ΔΔCt^ method [26].

### Western blot analysis

Total proteins in treated ATDC5 cells or mice knee joints cartilage tissues were isolated using M-PER TM Mammalian Protein Extraction Reagent (Thermo Fisher Scientific, Waltham, MA, USA). BCA TM Protein Assay kit (Thermo Fisher Scientific, Waltham, MA, USA) was applied for the quantification of total proteins. 25 μg of protein samples were separated by sodium dodecyl sulfate–polyacrylamide gel electrophoresis (SDS-PAGE), and transferred to PVDF membranes (Millipore, Billerica, MA, USA). After blocking with 5% non-fat milk for 1 h at room temperature, these membranes were incubated with the following primary antibodies (Abcam, Cambridge, UK) at 4 °C overnight: cleaved-caspase-3 (ab32042, 1:500), p62 (ab91526, 1:1000), Bcl-2 (ab59348; 1:1000), LC3B (ab48394; 1:400), Bax (ab7902; 1:500), SIRT1 (ab12193; 1:2000) and β-actin (ab6276, 1:5000). After washing three times with PBS, the membranes were incubated with the secondary antibody of horseradish peroxidase (HRP)-conjugated goat anti-rabbit IgG (ab205718, 1:2000, Abcam) for 2 h at room temperature. Signals of proteins were captured using Bio-Rad ChemiDocTM XRS system (Bio-Rad Laboratories, Hercules, CA, USA) and the intensities of proteins were quantified using Quantity One software (Bio-Rad Laboratories, Hercules, CA, USA).

### Cell viability assay

3-(4, 5-dimethylthiazol-2-yl)-2, 5-diphenyl-tetrazolium bromide (MTT) assay was applied to detect the cell viability. Cells were placed into the 96-well plate at a density of 5 × 10^3^ cells/well and cultured for 24 h. After incubation in serum-free DMEM/F12 medium for 24 h, the MTT solution (5 mg/ml, 20 μl) was added to each well and incubated for 4 h in a culture environment with 5% CO_2_ at 37 °C. Then, the culture medium was removed and 100 μl DMSO was supplemented to each well for 10 min. Finally, the optical absorbance at 590 nm was measured by a spectrophotometer (Thermo Fisher Scientific, Waltham, MA, USA). All assays were performed in three triplicates.

### Cell apoptosis analysis

Annexin V-FITC/PI apoptosis detection kit (Beijing Biosea Biotechnology, Beijing, China) was applied to detect the apoptotic cells. Briefly, ATDC5 cells were seeded into 6-well plate with 3 × 10^4^ cells per well and exposure to different treatment or transfection. The treated ATDC5 cells were then washed twice with cold PBS and re-suspended in buffer. Subsequently, cells were stained using 10 μl Annexin V-FITC/PI solution for 15 min in darkness at room temperature. Finally, the stained cells were subjected to flow cytometry analysis using Guava EasyCyte flow cytometer (Beckman Coulter, Fullerton, CA, USA), and the data were analyzed by the FlowJo software (Treestar, Ashland, OR, USA).

### Enzyme linked immunosorbent assay (ELISA)

For detecting the IL-6, TNF-α and leptin level in serum in vivo, blood samples of six mice per group were collected from the abdominal aorta, centrifuged at 1000 × *g* for 15 min, and then stored at − 80 °C. For in vitro assays, culture supernatant was obtained from 24-well plates after the indicated treatment. The levels of IL-6, TNF-α, PEG-2 and leptin were measured by using ELISA Kits (Abcam, Cambridge, MA) according to the manufacturers’ instructions.

### Dual luciferase reporter assays

Online bioinformatic tools StarBase 2.0 (http://starbase.sysu.edu.cn/), RNA hybrid (https://bibiserv.cebitec.uni-bielefeld.de/rnahybrid/) and DIANA tools (http://carolina.imis.athena-innovation.gr/diana_tools/web/) were used to predict the interactions of KCNQ1OT1 and miR-128-3p. The TargetScan (http://www.targetscan.org/vert_71/), miRDB (http://mirdb.org/) and RNA hybrid (https://bibiserv.cebitec.uni-bielefeld.de/rnahybrid/) were used to predict the binding sites within miR-128-3p and SIRT1. The sequences of KCNQ1OT1 and SIRT1 3’-UTR that included the wild-type and mutant-type binding sites of miR-128-3p were subcloned into a pGL3 vector (Promega, Madison, WI) to create the luciferase reporter vectors WT- KCNQ1OT1, MUT- KCNQ1OT1, WT- SIRT1 and MUT- SIRT1, respectively. Then, HEK-293 cells were transfected with NC mimic or miR-128-3p mimic along with the constructed luciferase reporter vectors by using Lipofectamine® 2000. After 48 h transfection, the relative luciferase activity was measured by a Dual-Luciferase Reporter Assay System (Promega, Madison, WI, USA). Renilla signals were normalized to firefly signals. All experiments were performed in triplicate.

### RNA immunoprecipitation (RIP) assay

RNA immunoprecipitation was performed using a Magna RIP RNA-Binding Protein Immunoprecipitation Kit (Millipore, USA). Briefly, ATDC5 cells were lysed in complete RIPA buffer containing a protease inhibitor cocktail and RNase inhibitor. Then, the lysates were precleared by centrifugation. The supernatants were incubated with RIP buffer supplemented with Anti-Ago2 antibody or negative control Anti-IgG beads (Millipore) overnight. After purifying the immunoprecipitated RNA in the magnetic beads, qRT-PCR analysis was subjected to detect the relative enrichment of KCNQ1OT1 and miR-128-3p.

### Double-labeled adenovirus mRFP-GFPLC3 transfection and autophagy detection

ATDC5 cells were plated and cultured in confocal dishes for 4 days, and then transfected with mRFP-GFP-LC3 lentivirus (Han Heng Biology, China) according to the manufacturer’s protocol. After transfection, cells were washed with PBS twice and then fixed with 4% paraformaldehyde. Fluorescent images were observed by a laser confocal microscopy (Zeiss, Oberkochen, Germany, LSM 510). The number of yellow spots (overlay of mRFP and GFP) represents autophagic bodies and red spots (mRFP alone) represent autophagic lysosomes.

### Transmission electron microscope (TEM)

After treatment, cells were digested with trypsin and centrifuged to obtain the cell masses. After removing the supernatant, cells were washed with PBS and centrifuged at 1500 r/min^−1^ at 4 °C for 5 min. The cell pellet was fixed with a precooled 2% glutaraldehyde solution at 4 °C for 2 h, and then post-fixed in 1% osmium tetroxide at room temperature for 2 h. After that, the cells were stained with 2% uranyl acetate solution for 2 h, dehydrated in 50%, 70%, 90% and 100% acetone, and then embedded in epoxy resin. The embedded block was cut into slices using ultramicrotome. After staining with saturated uranyl acetate and lead citrate, the slices were observed under a HITACHI, H-7500 Transmission Electron Microscope (HITACHI, H-7500, Japan).

### Statistical analysis

The data is represented as mean ± standard error of mean (S.E.M.). The body weight was analyzed with a two-way analysis of variance (two-way ANOVA) followed by Dunnett’s test. The other comparisons were conducted using one-way ANOVA or Student’s test. Pearson’s correlation analysis was used to assess the correlation between KCNQ1OT1 and miR-128-3p expression. A significant difference was judged to exist at a level of *P* < 0.05.

## Results

### Effects of Sal B on reduction of body weight and fat pad

**Fig. 1:**
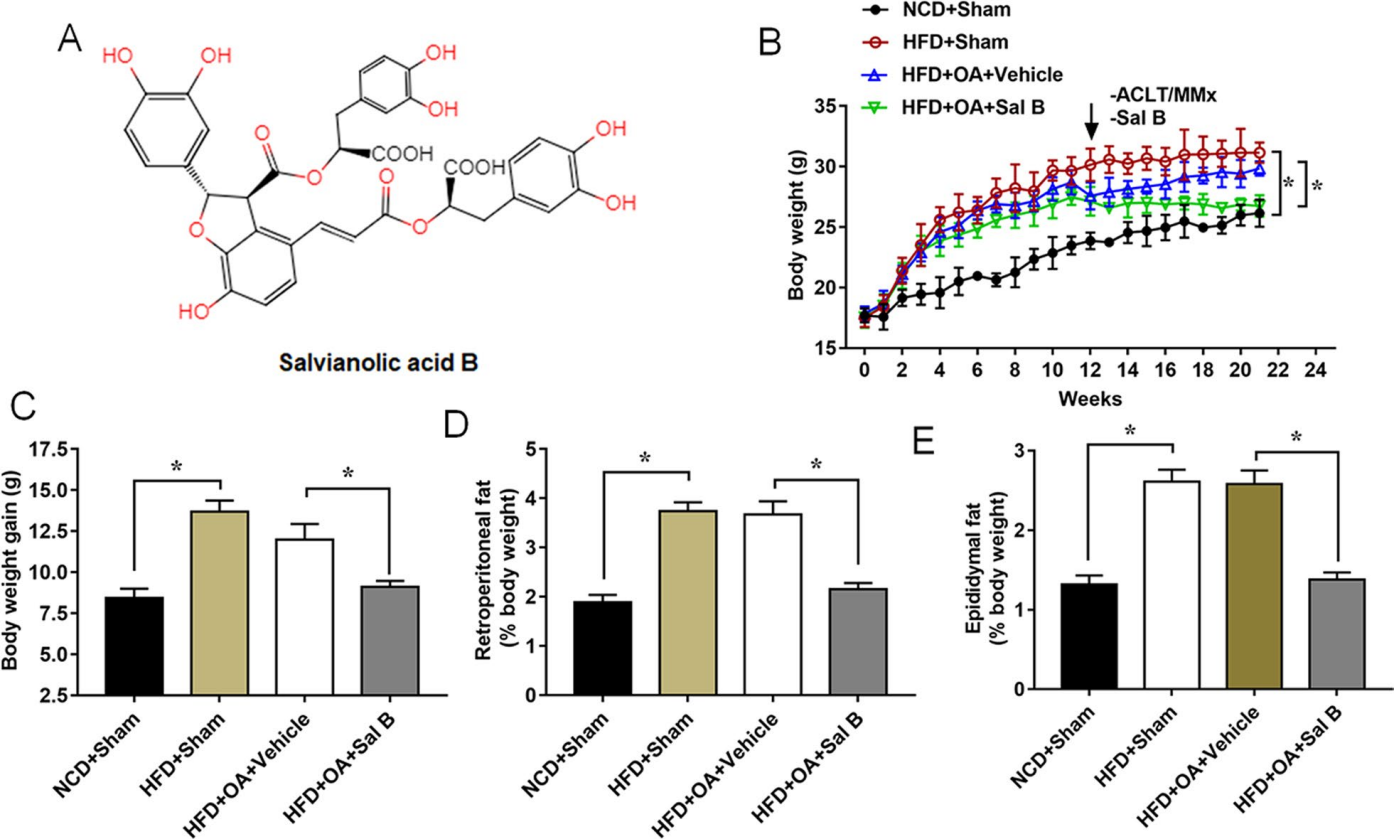
Effects of Salvianolic acid B (Sal B) on body weight and fat pad weight. **A** Chemical skeleton structure of Sal B. **B** Body weights were measured weekly for 21 weeks. **C** Body weight gain obtained from the difference between the body weights during the 21 weeks. Retroperorenal fat (**D**) and Epididymal fat (**E**) were measured after 21 weeks. Male C57BL/6 mice were divided into four groups (n = 6 per group). Data are expressed as means ± SEM. Student’s t test was used for the comparison between 2 groups in this study. *P < 0.05

The Sal B chemical structure was shown in Fig. 1A, and the effect of Sal B on body weight changes was shown in Fig. 1B and Additional file 1: Table S1. From the 12th week, the mice of Sal B group began to show a slower growth compared with the vehicle group (Fig. 1B). The body weight of mice in the vehicle group gained an increase of 11.99 ± 0.96 g from 1th week to 20th weeks, while that of mice in Sal B group (9.2 ± 0.5 g) was significantly less than the vehicle group (Fig. 1B). Moreover, the percentages of retroperorenal fat and epididymal fat in body weight were significantly increased in HFD group compared with NCD group, while 25 mg/kg Sal B treatment decreased the percentages of retroperorenal fat and epididymal fat in body weight (Fig. 1C–E, Additional file 1: Table S1). These data indicated that the administration of Sal B inhibited HFD-induced weight gain in mice that underwent the ACLT/MMx.

### Sal B reduces the development of osteoarthritis in obese mice

**Fig. 2:**
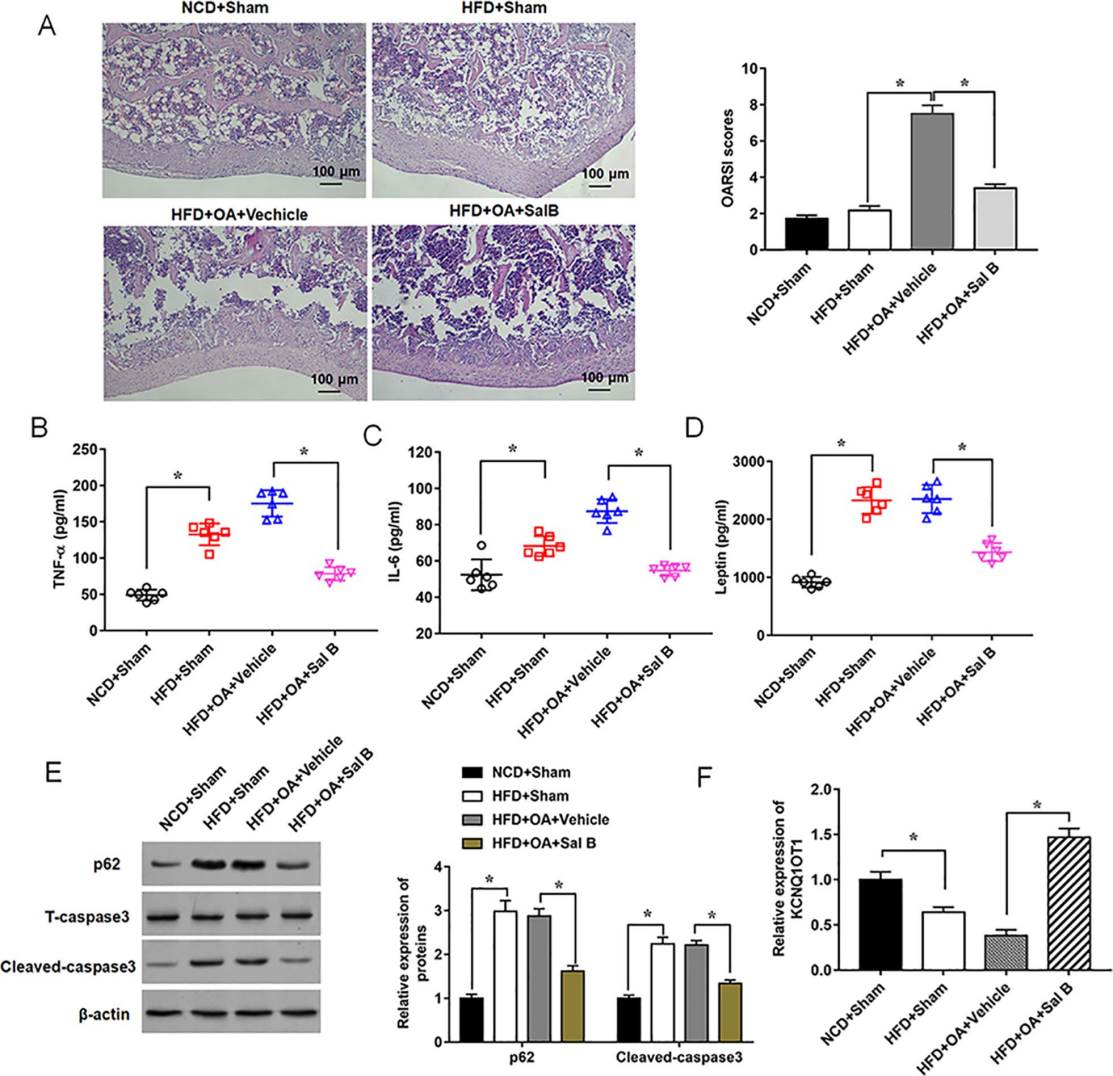
Sal B inhibits histological changes of knee joints and inflammatory response in mice fed with HFD. **A** Two knee joints per mouse with OA were collected after 21 weeks and stained with H & E (original magnification, 100 ×), Scale bars = 100 μm. **B**–**D** Serum levels of TNF-α, IL-6 and leptin were measured by ELISA. **E** Relative expression levels of p62 and Cleaved-caspase3 were detected by Western blot analysis. **F** Expression of KCNQ1OT1 was determined by RT-qPCR. n = 6. Data are expressed as means ± SEM. Student’s t test was used for the comparison between 2 groups in this study. *P < 0.05

At the end of the experiments, the mice were euthanized and the knee joint specimens were collected. The joint sections were stained with hematoxylin and eosin stain to observe the morphological changes in different groups. The results showed the mild damage of cartilage structure in the HFD group and OA groups, while an improvement was observed in Sal B group (Fig. 2A). The OARSI score of the HFD group was greater than the scores of the NCD group and they were reversed by Sal B treatment, which were consistent with the results of histologic analysis (Fig. 2A). Moreover, the serum levels of TNF-α, IL-6 and leptin were significantly increased in HFD group and in OA group, while these increases were weakened by 25 mg/kg Sal B treatment (Fig. 2B–D, Additional file 1: Table S2). Western blot results showed that the expression of p62 and Cleaved-caspase3 obviously were decreased in Sal B group compared with the vehicle group (Fig. 2E). More importantly, KCNQ1OT1 was downregulated in mice fed with HFD, as well as in OA mice, while Sal B administration notably increased the expression level of KCNQ1OT1 (Fig. 2F). Thus, our results indicated that Sal B can alleviate the development of obesity-related osteoarthritis in mice.

### Sal B protects ATDC5 cells against PA-mediated inflammation and apoptosis, and increases cell autophagy

**Fig. 3:**
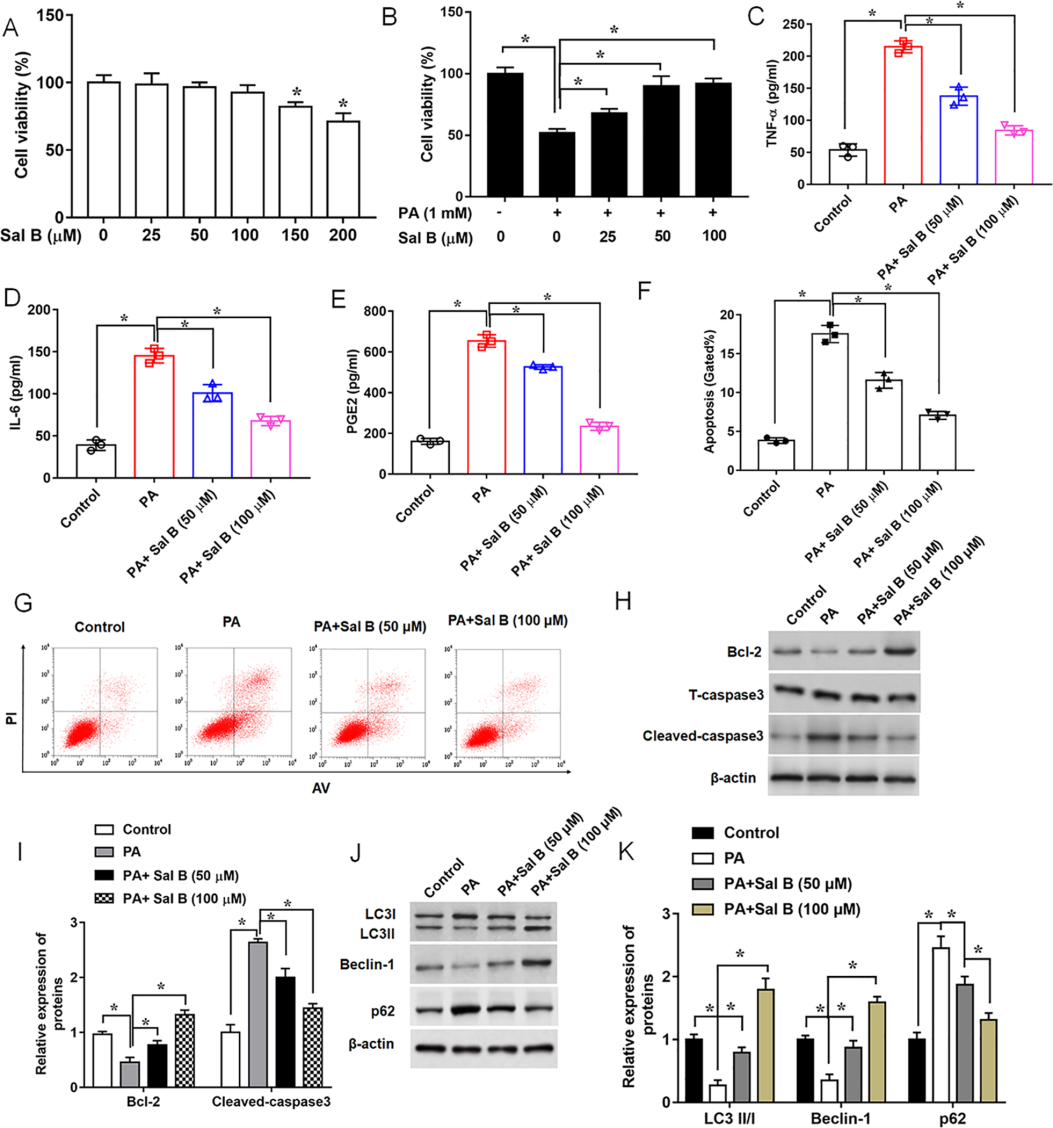
Sal B promotes chondrocyte autophagy, and reduces PA-induced apoptosis and inflammation. **A** Viability of ATDC5 cells was detected by MTT assay following the treatment of different concentrations of Sal B for 24 h. **B** Cell viability was measured in cells treated with Sal B (25 μM, 50 μM or 100 μM) followed by the treatment of PA (500 μM, 24 h). **C**–**E** Concentrations of pro-inflammatory cytokines in the culture supernatant, **F** and **G** Apoptotic ratio, **H** and **I** expression levels of apoptosis-related proteins, and **J** and **K** expression levels of autophagy-related proteins were assayed, after ATDC5 cells were in turn treated with Sal B (50 μM or 100 μM) for 24 h, and 500 μM PA for another 24 h. n = 3. Data are expressed as means ± SEM. One-way ANOVA was used for the comparison in this study. *P < 0.05

In this study, we detected the effects of Sal B on ATDC5 cell viability. After administrated ATDC5 cells with a series of concentrations of Sal B for 24 h, the viability of cells was detected by MTT assay to evaluate cytotoxicity of Sal B. The results revealed that Sal B at the concentrations < 150 μM obviously decreased the cell viability (Fig. 3A). Then, cells were administered with Sal B (25 μM, 50 μM or 100 μM) after the stimulus of PA (500 μM, 24 h), and the results showed that Sal B administration significantly alleviated PA-induced ATDC5 cells injury in a dose-dependent manner (Fig. 3B). Then, the concentrations of pro-inflammatory cytokines (TNF-α, IL-6 and PGE2) were observed by ELISA. We found that the secretion of TNF-α, IL-6 and PGE2 were increased in the PA- stimulated ATDC5 cells compared with the control group, which were decreased after the Sal B treatment (Fig. 3C–E). We next detected cell apoptosis rate of ATDC5 cells by flow cytometry. The results revealed that PA induced a significant cell apoptosis rate in ATDC5 cells (Fig. 3F, G). These observations were coupled with the down-regulated expression of Bcl-2 and the cleavage of caspase-3, suggesting PA significantly damaged ATDC5 cells. While Sal B strikingly alleviated PA-induced cell apoptosis (Fig. 3H, I). Additionally, Western blot demonstrated that PA suppressed cell autophagy in ATDC5 cells, as the expression levels of LC3II/I ratio and Beclin-1 were decreased, and the expression of p62 was enhanced by PA stimulation. However, the suppressive effects of PA on cell autophagy were attenuated by Sal B treatment (Fig. 3J, K). To provide a more efficient method for detection of autophagy, we transfected ATDC5 with mRFP-GFP-LC3 virus and observed autophagy flux using laser confocal microscopy. Autophagosomes were labeled red and green (yellow fluorescence), whereas autophagic lysosomes were labeled red. Our results showed that stimulus of PA decreased autophagy level, while the Sal B exhibited higher autophagy level than the PA group in a dose-dependent manner (Additional file 2: Fig. S1). Thus, Sal B at a concentration of 100 μM was used in the subsequent experiments. Collectively, these data revealed that treating ATDC5 cells with Sal B effectively alleviated PA-induced cell death and inflammatory injury and enhanced cell autophagy.

### Knockdown of KCNQ1OT1 weakens the therapeutic functions of Sal B in PA-induced ATDC5 cell injury

**Fig. 4:**
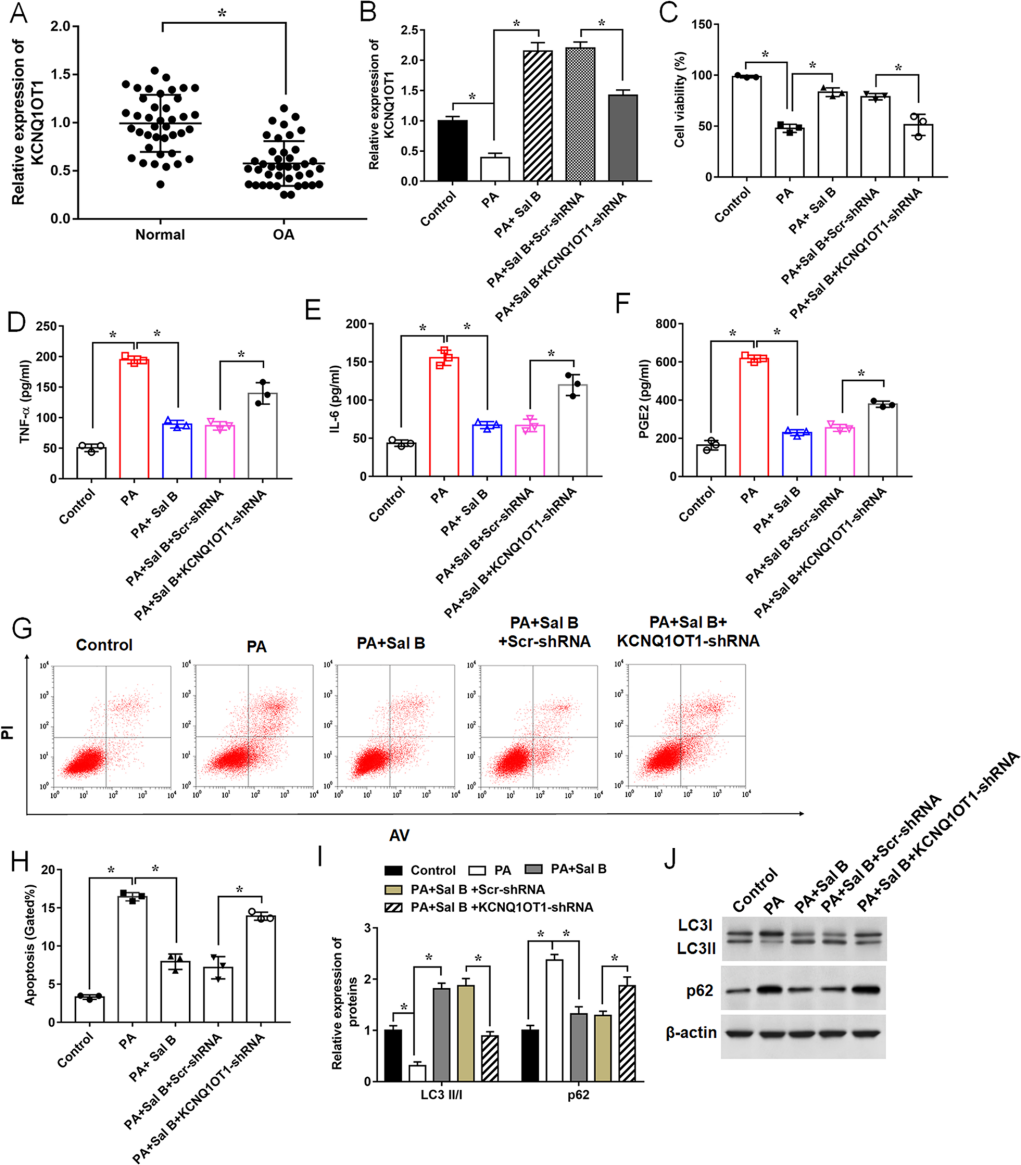
Effects of KCNQ1OT1 knockdown on the protective functions of Sal B in PA-induced ATDC5 cells. **A** Relative expression of KCNQ1OT1 in cartilage tissues obtained from normal group and OA group was determined by RT-qPCR. **B** Expression of KCNQ1OT1, **C** Cell viability, **D**–**F** Concentrations of pro-inflammatory cytokines in the culture supernatant, **G** and **H** Apoptotic ratio, and **I** and **J** expression levels of autophagy-related proteins were determined, after ATDC5 cells were in turn transfected with KCNQ1OT1 shRNA or Scr-shRNA, treated with 100 μM Sal B for 24 h, and treated with 500 μM PA for another 24 h. n = 3. Data are expressed as means ± SEM. Student’s t test and one-way ANOVA were used for the comparison in this study. *P < 0.05

Previous study demonstrated that resveratrol, which possesses similar functions with Sal B in antioxidant and anti-inflammation, could relieve osteolysis through the upregulation of KCNQ1OT1 [10]. Therefore, to further explore the therapeutic effects of Sal B on PA-induced chondrocyte injury, we detected the KCNQ1OT1 expression in OA tissues by RT-qPCR. The results showed that KCNQ1OT1 expression was significantly downregulated in OA tissues compared with normal cartilage tissues (Fig. 4A). Similarly, KCNQ1OT1 expression was downregulated by PA stimulus while upregulated by Sal B treatment in ATDC5 cells, indicating KCNQ1OT1 as a downstream effector of Sal B (Fig. 4B). To verify this hypothesis, we explored whether the therapeutic effects of Sal B on PA-induced chondrocyte damage could be altered by knockdown of KCNQ1OT1. As shown in Fig. 4C–E, the results suggested that the downregulation of KCNQ1OT1 significantly weakened the suppressive effects of Sal B on PA-induced cell damage, as cell viability (Fig. 4C) was decreased, concentrations of pro-inflammatory cytokines (Fig. 4D–F) were increased, and cell apoptosis rate (Fig. 4G, H) was enhanced in ATDC5 cells transfected with KCNQ1OT1 shRNA. Moreover, the effects of Sal B on the expression of LC3II/I ratio and p62 were attenuated by KCNQ1OT1 shRNA (Fig. 4I, J), indicating Sal B could promote cell autophagy by upregulating the level of KCNQ1OT1 expression. Furthermore, we found Sal B treatment significantly increased the number of autophagosomes, while KCNQIOT1 knockdown weakened the promoting effect of Sal B on the number of autophagosomes using TEM (Additional file 3: Fig. S2). Therefore, the above results suggested that Sal B protected ATDC5 cells against PA-induced damage through the upregulation of KCNQ1OT1.

### KCNQ1OT1 directly regulates the miR-128-3p/ SIRT1 axis in ATDC5 cells

**Fig. 5:**
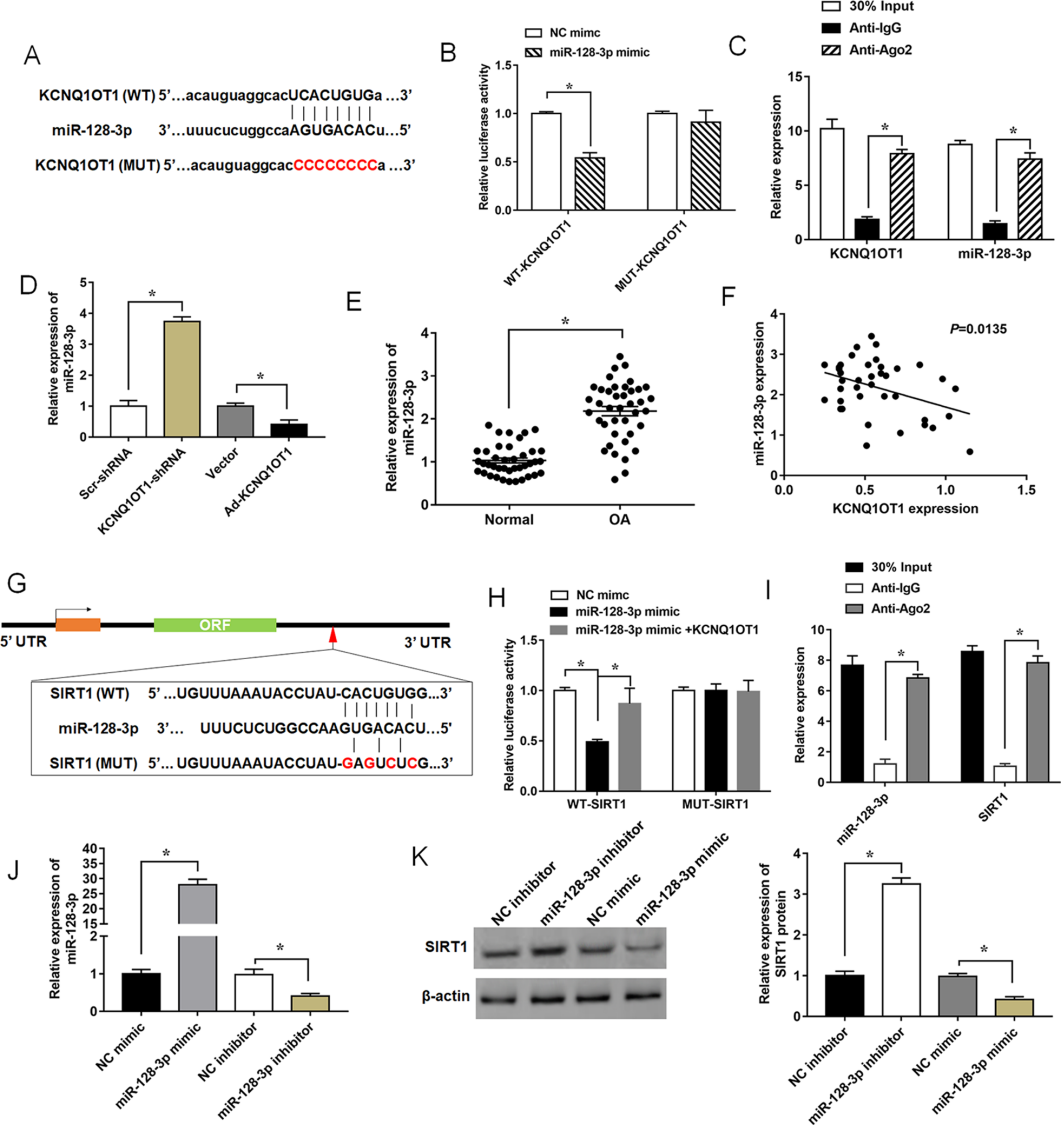
KCNQ1OT1 is a ceRNA of miR-128-3p in the regulation of SIRT1 expression. **A** The binding sites of KCNQ1OT1 and miR-128-3p were predicted by bioinformatics analysis. **B** Dual luciferase reporter assay was performed to detect the luciferase activity of HEK-293 T cells co-transfected with miR-128-3p mimic and luciferase reporter vectors containing WT- or MUT- KCNQ1OT1 3’UTR. **C** The enrichment of KCNQ1OT1 and miR-128-3p in IgG or Ago2 immunoprecipitates detected by RIP and RT-qPCR. Anti-IgG served as a negative control, and 30% Input (cells extracts) served as a positive control. **D** Relative expression levels of miR-128-3p in ATDC5 cells transfected with KCNQ1OT1 shRNA, Ad-KCNQ1OT1 or their negative controls. **E** Expression of miR-128-3p in cartilage tissues obtained from normal group and OA group was determined. **F** The correlation analysis between KCNQ1OT1 and miR-128-3p was determined by spearman analysis. **G** The putative binding sites between miR-128-3p and SIRT1. **H** and **I** Dual-luciferase reporter gene assay and RIP analysis were performed to verify the correlation of miR-128-3p and SIRT1. Anti-IgG served as a negative control, and 30% Input (cells extracts) served as a positive control. **J** and **K** Expression levels of miR-128-3p and SIRT1 protein were detected in ATDC5 cells transfected with miR-128-3p mimic, miR-128-3p inhibitor and their negative control. Data are expressed as means ± SEM. Student’s t test was used for the comparison between 2 groups in this study. *P < 0.05

LncRNAs exert their biological effects by functioning as competing endogenous RNAs to suppress miRNA expression, thus influencing the expression of miRNA downstream targets [17]. In this study, we predicted the binding sites of KCNQ1OT1 and miR-128-3p by using online bioinformatic tools (Fig. 5A, Additional file 4). Then, the results of the luciferase reporter assay revealed that miR-128-3p mimic significantly decreased the luciferase activity of WT- KCNQ1OT1 3’-UTR reporter vectors, while no evident effect was found on luciferase activity of MUT- KCNQ1OT1 3’-UTR reporter vectors (Fig. 5B, Additional file 5). To confirm whether miR-128-3p could bind KCNQ1OT1, the RIP assay demonstrated an enrichment of KCNQ1OT1 and miR-128-3p in the Ago2 pellet compared with Anti-IgG (Fig. 5C). RT-qPCR revealed that miR-128-3p was significantly increased by KCNQ1OT1 deficiency and decreased by KCNQ1OT1 overexpression (Fig. 5D). In addition, RT-qPCR analysis also illustrated miR-128-3p was significantly upregulated in cartilage tissues obtained from normal group and OA group (Fig. 5E). And the Spearman correlation analysis showed there was a significant negative correlation between expression of KCNQ1OT1 and miR-128-3p in OA cartilage tissues (Fig. 5F). All these data indicated that KCNQ1OT1 could interact with miR-128-3p, and negatively regulated the expression of miR-128-3p.

To further explore the underlying mechanism of miR-128-3p that was a target of KCNQ1OT1 on OA development, we predicted the downstream targets of miR-128-3p by online bioinformatics analysis and found that miR-128-3p could bind to the 3’-UTR of SIRT1 (Fig. 5G, Additional file 4). Sirt1 is critical for normal skeletal development and homeostasis by regulating chondrocytes and bone cells actions, and play vital role in metabolism, including fat storage, gluconeogenesis, fatty acid oxidation, lipogenesis, insulin secretion, and inflammation [3]. Accumulating evidence demonstrated that SIRT1 could prevent cartilage degeneration by activating autophagy [29, 34], and suppress adipogenesis to ameliorate obesity [21]. The dual luciferase reporter assay revealed that miR-128-3p significantly decreased the luciferase activity of the WT-SIRT1 group, but overexpressed KCNQ1OT1 weakened this inhibitory effect. While the luciferase activity displayed no significant changes in luciferase reporter vector containing MUT-SIRT1 3’-UTR compared the control group (Fig. 5H, Additional file 5). We next conducted the RIP assay to confirm the association between miR-128-3p and SIRT1, and the results showed that miR-128-3p and SIRT1 expression were both enriched in Anti-Ago2 pellet (Fig. 5I). Then, we detected the expression of miR-128-3p in ATDC5 cells after transfection of miR-128-3p mimic, miR-128-3p inhibitor or their negative control (Fig. 5J). The expression of SIRT1 was promoted by the knockdown of miR-128-3p, and suppressed by the overexpression of miR-128-3p in ATDC5 cells (Fig. 5K). Collectively, these results suggested that KCNQ1OT1 was involved the OA development via miR-128-3p/SIRT1 axis.

### miR-128-3p overexpression or SIRT1 knockdown can reverse the effects of Sal B on apoptosis, inflammation, and autophagy in PA-stimulated ATDC5 cells

**Fig. 6:**
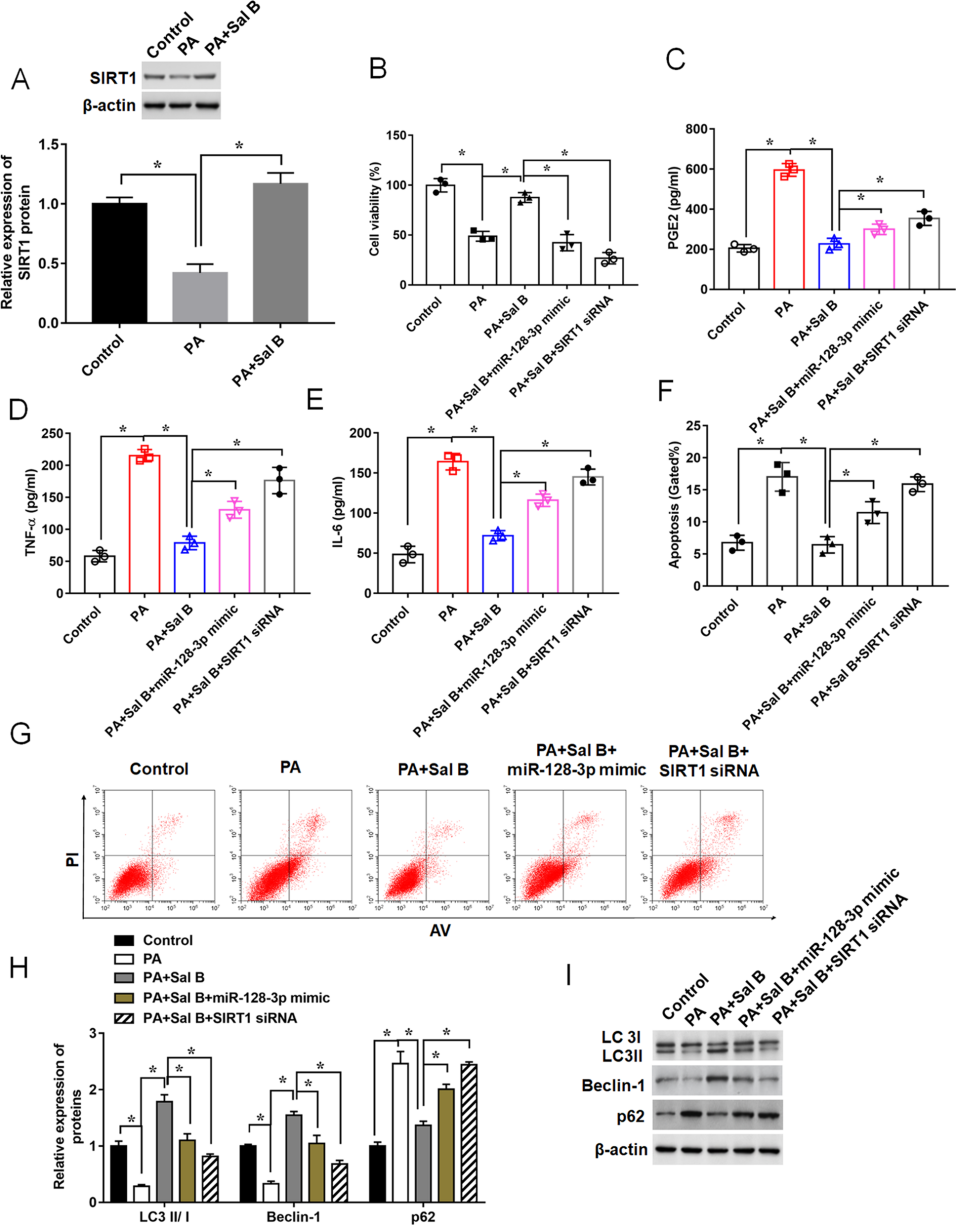
Influences of miR-128-3p overexpression or SIRT1 silence on the effects of Sal B on PA-mediated ATDC5 cells. ATDC5 cells were transfected with miR-128-3p mimic or SIRT1 siRNA, and treated with Sal B (100 μM, 24 h) followed by the treatment of PA (500 μM, 24 h). **A** Relative expression of SIRT1 was determined by Western blot assay. **B** Cell viability, **C**–**E** Concentrations of pro-inflammatory cytokines in the culture supernatant, **F** and **G** Apoptotic ratio, and **H** and **I** expression levels of autophagy-related proteins were measured by MTT assay, ELISA assay, Flow cytometry analysis and Western blot, respectively. n = 3. Data are expressed as means ± SEM. Student’s t test was used for the comparison between 2 groups in this study. *P < 0.05

Then, we found that PA significantly decreased the expression of SIRT1, while Sal B weakened this inhibitory effect (Fig. 6A). To verify the above results that Sal B exerts its role in alleviating the development of arthritis via the miR-128-3p/SIRT1 axis, we overexpressed miR-128-3p or knocked down SIRT1 in ATDC5 cells followed by the treatment with PA and Sal B. MTT assay indicated that miR-128-3p overexpression or SIRT1 knockdown could weakened the effects of Sal B on cell viability in PA pretreating ATDC5 cells (Fig. 6B). Then, we detected the concentrations of pro-inflammatory cytokines in the culture supernatant. We found that miR-128-3p overexpression enhanced inflammatory response compared with the Sal B and PA group, and knockdown of SIRT1 displayed similar results (Fig. 6C–E). Similarly, transfection of miR-128-3p mimic or SIRT1 siRNA could reduce the inhibitory effect of Sal B on cell apoptosis, and promoted the apoptosis ratio (Fig. 6F, G). SIRT1 is verified to be involved in autophagy process to play its positive roles in OA [3, 29]. In our study, the results showed that miR-128-3p overexpression significantly suppressed the Sal B-mediated cell autophagy, which was consistent with the result of SIRT1 knockdown (Fig. 6H, I). Thus, all above data indicated that miR-128-3p/SIRT1 axis was involved in the effects of Sal B treatment on apoptosis, inflammation, and autophagy in PA-stimulated ATDC5 cells.

### Sal B affects PA-mediated autophagy and apoptosis in chondrocytes by suppressing JAK2 / STAT3 pathway

**Fig. 7:**
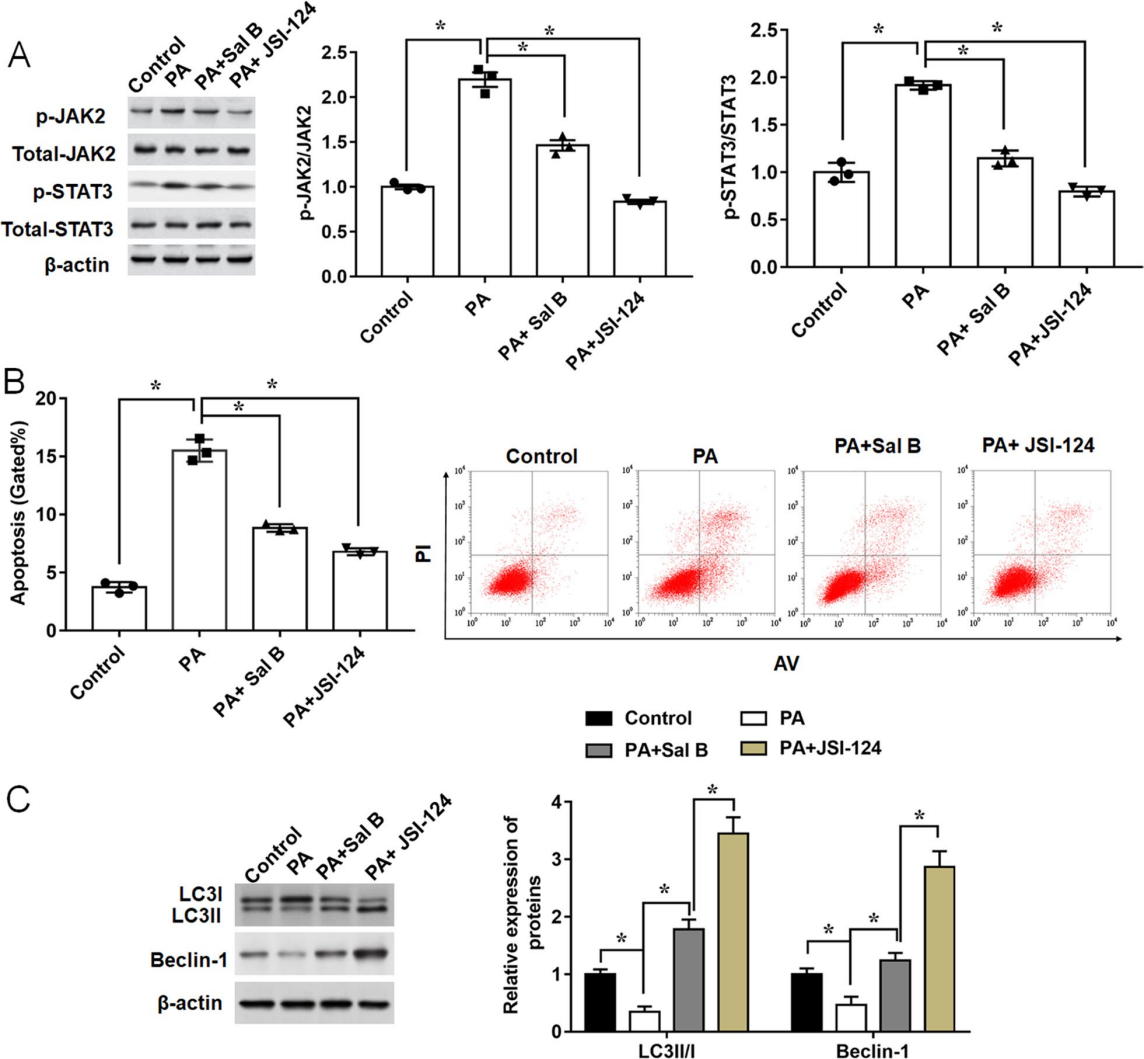
JAK2/STAT3 pathway inhibition weakens the effects of Sal B on PA-mediated ATDC5 cell autophagy and apoptosis. ATDC5 cells were treated with Sal B (100 μM, 24 h), followed by the treatment of PA or JSI-124 (10 μM, 24 h). **A** The protein levels of p-JAK2, JAK2, pSTAT3, STAT3 were determined by Western blot. **B** Apoptotic ratio was detected by Flow cytometry analysis. **C** Relative expression levels of autophagy-related proteins were measured by Western blot. n = 3. Data are expressed as means ± SEM. One-way ANOVA was used for the comparison between 2 groups in this study. *P < 0.05

Janus Kinase 2 (JAK2)/Signal Transducers and Activators of Transcription 3 (STAT3) signaling is regarded to be involved in the regulation of cellular responses to inflammatory and apoptosis in chondrocytes [45]. We used 10 μM JSI-124, a specific inhibitor of the signaling pathway involving the JAK2/ STAT3 signaling molecules to further investigate the mechanisms of Sal B. As shown in Fig. 7A, expression levels of p-JAK2 and p-STAT3 were elevated by PA treatment. However, Sal B significantly decreased the phosphorylation levels of JAK2 and STAT3 induced by PA, which were consistent with results of the inhibitor JSI-124 (Fig. 7A). Meanwhile, cell apoptosis presented the same trend (Fig. 7B). Apart from that, the expression of autophagy related proteins showed significant increases in JSI-124 treatment group (Fig. 7C). Therefore, our results indicated that Sal B could block the JAK2/STAT3 pathway to alleviate PA-induced chondrocytes damage.

## Discussion

Obesity-related OA, a subtype of metabolic OA, has been suggested to be caused by mechanical contribution to joint load and chronic low-grade inflammation in obese individuals [36]. Altogether, the presented results suggested that Sal B might function as a new therapeutic drug for the treatment of obesity-related OA. Emerging evidence indicated that many Chinese herbs played important roles in the treatment of OA because of their rich natural ingredients. Sal B, as the most active constituent of water-soluble polyphenolic acid substances in *Salvia miltiorrhiza*, displayed significantly roles in reducing obesity and obesity-related metabolic disorders [28]. Due to the complexity of the pathogenesis and pathological environment of obesity-related OA, it is essential to carry out the animal model experiments before the clinical application of the in vitro experimental results. Through the animal experiments, the changes in the cellular and molecular levels in the joint tissues can be observed after the treatment of obesity-related OA with Sal B, which lays the foundation for the successful translation of new drugs for the treatment of obesity-related OA into clinical practice. Thus, we investigated the effect of Sal B on OA procession in the OA mice model fed with HFD. Our results showed that Sal B decreased body weight induced by HFD, which was consistent with the results of Zhao et al. [46] that Sal B notably improved glycolipid metabolism and reduced body weight in HFD-mediated obese mice. Therefore, all these results suggested that Sal B might function as a new therapeutic drug for the treatment of obesity-related OA.

In the past decades, emerging evidence has demonstrated that lncRNAs function as the Sal B therapy targets through serving as competing endogenous RNAs (ceRNAs) to sponge miRNAs, consequently modulating the downstream targets of miRNAs. For example, Yu et al. found that lincRNA-p21 expression was upregulated in HSCs after Sal B treatment, and then inhibited the Wnt/β-catenin pathway in activated hepatic stellate cells via sponging microRNA-17-5p [40]. In this study, we found that Sal B upregulated the expression of KCNQ1OT1 in OA mice model and PA-induced ATDC5 cells, suggesting that KCNQ1OT1 might be a momentous target of Sal B. KCNQ1OT1 was demonstrated to play an important role in OA progression via sponging has-miR-1202-ETS1 interaction [23, 24]. KCNQ1OT1 expression was downregulated in osteoarthritic chondrocytes, and upregulation of KCNQ1OT1 significantly enhanced the viability of osteoarthritic chondrocytes, inhibited cell apoptosis, and reduced the release of inflammatory cytokines and metal matrix enzymes through regulating the miR-218-5p/PIK3C2A axis [25]. A recent study revealed that KCNQ1OT1 was highly expressed in the cartilage tissues of patients with OA and OA cells treated with LPS, and knockdown of KCNQ1OT1 stimulated cell viability, and suppressed the inflammation and degradation of the extracellular matrix (ECM) in OA cells by mediating the miR-211-5p/TCF4 axis [2]. Thus, KCNQ1OT1 functioning as a ceRNA could play different roles in OA progression. However, the role of KCNQ1OT1 by interacting with miR-128-3p in the OA progression remains largely unknown. In this study, luciferase reporter assay and RIP assays showed that KCNQ1OT1 interacted with miR-128-3p, and negatively regulated the expression of miR-128-3p, thus regulating cell apoptosis, autophagy, and inflammatory response in ATDC5 cells. More importantly, RNA-Seq data showed that 234 lncRNAs were differentially expressed in white adipose tissue under Sal B treatment, and several differentially expressed lncRNA may participate in lipid metabolism and sugar metabolism [4]. Apart from Sal B, resveratrol was demonstrated to accelerate osteoblast differentiation by regulating lncRNA KCNQ1OT1 via the activation of Wnt/β-catenin pathway [10]. Moreover, since Sal B is one of the phenolic acids isolated from Salvia miltiorrhiza, we speculate that other phenolic acids that possess anti-inflammatory and antioxidant properties, such as Lithospermic acid [15], Rosmarinic acid [13] and Caffeic acid [14], could improve obesity-related OA through the KCNQ1OT1/miR-128-3p/SIRT1 pathway. However, more experiments still need to be conducted to confirm. Thus, all above evidence suggested that Sal B may exert its therapeutic function by upregulating KCNQ1OT1 in PA-stimulated chondrocyte via the miR-128-3p/SIRT1 axis, indicating KCNQ1OT1 may serve as a therapeutic target for OA.

Previous studies reported that Sal B exerted protective effects in several diseases through its anti-inflammatory activity [18, 47]. For example, Sal B dose-dependently suppressed IL-β induced the expression of iNOS, COX-2, MMP-13 and ADAMTS-5 via the inhibition of NF-κB p65 signaling in human osteoarthritis chondrocytes [27]. Sal B could effectively attenuate inflammation through activating the Nrf2-mediated antioxidant defense system using a C57BL/6 mouse model [33]. Similarly, we found that the secretion of TNF-α, IL-6 and PGE2 were increased in the PA-treated ATDC5 cells compared with the control group, which was alleviated after the Sal B administration. However, KCNQ1OT1 knockdown reversed the inhibitory effects of Sal B on inflammatory response. On the other hand, in vivo experiments indicated that Sal B significantly suppressed inflammatory response in a mouse OA model, which was consistent with the in vitro results. And Sal B promoted cellular activities and increased viable chondrocytes through directly stimulating the expression of SOX9, which suggested Sal B could be applicable to treatments for osteochondral damage repairs [38]. In this study, we observed that Sal B promoted chondrocytes viability, while the downregulation of KCNQ1OT1 weakened this effect.

In addition to the antioxidant and anti-inflammatory properties of Sal B, its protective effect can also be attributed to its ability to promote autophagy of chondrocytes. Autophagy is a major catabolic process of eukaryotic cells that degrades and recycles damaged macromolecules and organelles [6]. In cartilage homeostasis, the activation of chondrocyte autophagy can reduce the severity of OA [5]. Previous studies demonstrated that curcumin alleviated the development of OA through the activation of chondrocyte autophagy both in vivo and in vitro [22, 43]. By analyzing the expression of autophagy related genes in healthy and OA cartilage tissues, 20 autophagy related genes were downregulated in OA tissues, including LC3 and Beclin1 [44]. Similarly, our results showed that Sal B effectively increased the Beclin 1 and LC3 expression in PA-induced ATDC5 cells, which suggested Sal B may exhibit its protective effect on OA through activating autophagy. In chondrocytes, the NAD-dependent deacetylase SIRT1 reduction may result in chondrocyte hypertrophy and cartilage matrix loss. It was verified that SIRT1 directly activated autophagy in human chondrocytes to enhance chondrogenesis and the prevention of OA [3, 29]. Metformin activated autophagy, reduces apoptosis and mitigates cartilage degradation in mice with OA by the upregulation SIRT1 [34]. In the present study, SIRT1 that was verified as a target of miR-128-3p was downregulated in PA-induced ATDC5 cells, while Sal B treatment could significantly promote the level of SIRT1 expression. Subsequently, we found that cell apoptosis was decreased and autophagy was significantly enhanced after Sal B treatment, while SIRT1 knockdown reversed these results. Thus, Sal B activates chondrocytes autophagy and reduces chondrocyte apoptosis via the KCNQ1OT1/miR-128-3p/SIRT1 signaling pathways, in which KCNQ1OT1 acted as miR-128-3p sponges to regulate SIRT1 expression according to a ceRNA network.

In summary, our findings demonstrated that HFD fed significantly increased the incidence of knee OA, while Sal B administration partially alleviated the development of OA by the reduction of body weight, decreasing inflammatory response in OA mice model. Moreover, we observed that Sal B protects ATDC5 cells from PA-induced inflammatory and apoptotic injury by upregulating the KCNQ1OT1 expression, and thereby activating cell autophagy via miR-128-3p/SIRT1/ JAK2/STAT3 pathway. This study reveals the potential mechanism of Sal B in obesity-related OA, and further confirms the therapeutic value of Sal B in obesity-related diseases, thus providing a theoretical basis for clinical application of Sal B.

However, because of the small joints and thin cartilage tissue in mice, it is difficult to obtain materials and identify the degree of damage, which requires the research results to be verified in other animal models to reduce the one-sidedness of the conclusions and help us to better understand the therapeutic effects of Sal B on obesity-related OA. Furthermore, in the preparations with salvianolic acid B as the active ingredient, because of its unstable chemical structure, Sal B is easily degraded in aqueous solution, and various degradation products have complex pharmacological activities, leading to the reduced efficacy of SalB in clinical application. Thus, there are still some problems that need to be further explored on how to maintain its stability in the solution, such as investigating the selection of stability indicators, the mutual interference of the ingredients in the compound, the influence of excipients on stability and the reaction mechanism, etc. Obesity-related OA is a complex regulatory network, and what role Sal B plays in this regulatory network still needs to be explored before the clinical application of Sal B in the treatment of obesity-related OA.

## Supplementary Information

**Additional file 1.** Supplemental Tables.

**Additional file 2: Fig. S1.** Sal B treatment induced autophagy in PA-stimulated ATDC5 cells. Autophagic flux of mRFP-GFP-LC3-transfected ATDC5 cells revealed by laser confocal microscopy. Autophagosomes are labeled by red and green fluorescence (yellow spots), whereas autophagic lysosomes are labeled by red fluorescence (red spots). Magnification: × 1000.

**Additional file 3: Fig. S2.** Knockdown of KCNQ1OT1 weakened the promoting of Sal B on autophagy in PA-stimulated ATDC5 cells. Transmission electron micrographs of autophagosomes in ATDC5 cells. Magnification: × 8000.

**Additional file 4.** Prediction for the interactions of KCNQ1OT1 and miR-128-3p by different bioinformatic tools.

**Additional file 5.** Values of luciferase intensity in the Luciferase Reporter Gene Assays.

## Data Availability

The data and materials that support the findings of this study are available from the corresponding author upon reasonable request.

## Abbreviations

OA: Osteoarthritis
Sal B: Salvianolic acid B
lncRNA KCNQ1OT1: LncRNA KCNQ1 overlapping transcript 1
SIRT1: Sirtuin-1
HFD: High fat diet
JAK2: Janus kinase 2
STAT3: Signal transducers and activators of transcription 3
