# Supplementary material for: Salvianolic acid B activates chondrocytes autophagy and reduces chondrocyte apoptosis in obese mice via the KCNQ1OT1/miR-128-3p/SIRT1 signaling pathways
Source: Nutr Metab (Lond). 2022 Aug 3;19:53. doi: 10.1186/s12986-022-00686-0 (PMC9351265; doi:10.1186/s12986-022-00686-0)
Supplement: Supplementary file 1 — Additional file 1. Supplemental Tables. [file 12986_2022_686_MOESM1_ESM.docx]

**Supplemental Table 1. Effects of Sal B on body weight and fat pad weight**

| Group | Body weight gain  (g) | retroperorenal fat  (% body weight) | epididymal fat  (% body weight) |
| --- | --- | --- | --- |
| NCD+Sham | 8.44±0.57 | 1.89±0.15 | 1.32±0.11 |
| HFD+Sham | 13.68±0.69* | 3.74±0.18* | 2.61±0.15* |
| HFD+OA+Vehicle | 11.99±0.96^#^ | 3.67±0.27^#^ | 2.58±0.17^#^ |
| HFD+OA+Sal B  (7.5 mg/kg) | 10.54±0.49^#^ | 3.35±0.29^#^ | 2.47±0.09^#^ |
| HFD+OA+Sal B (12.5 mg/kg) | 9.97±0.64^#^ | 2.94±0.38^#^ | 2.24±0.17^#^ |
| HFD+OA+Sal B  (25 mg/kg) | 9.11±0.37^&^ | 2.15±0.13^&^ | 1.38±0.09^&^ |

NCD: normal control diet; HFD: high-fat diet. **P*<0.05 vs. NCD+Sham group; ^#^ *P*<0.05 vs. HFD+Sham group; ^&^*P*<0.05 vs. HFD+OA+Vehicle group.

**Supplemental Table 2. Effects of Sal B on the levels of TNF-α, IL-6 and Leptin in obesity-related OA**

| Group | TNF-α  (pg/ml) | IL-6  (pg/ml) | Leptin  (pg/ml) |
| --- | --- | --- | --- |
| NCD+Sham | 49.10±3.07 | 52.42±3.48 | 919.70±37.63 |
| HFD+Sham | 132.8±6.11* | 68.25±2.28* | 2331.00±93.39* |
| HFD+OA+Vehicle | 175.40±7.47^#^ | 87.49±2.68^#^ | 2355.00±99.84^#^ |
| HFD+OA+Sal B  (7.5 mg/kg) | 154.71±10.21^#^ | 79.85±5.47^#^ | 2197.10±115.60^#^ |
| HFD+OA+Sal B (12.5 mg/kg) | 138.65±9.58^&^ | 74.01±6.95^#^ | 1894.10±131.50^&^ |
| HFD+OA+Sal B  (25 mg/kg) | 78.40±3.77^&^ | 54.70±1.25^&^ | 1437.00±63.72^&^ |

NCD: normal control diet; HFD: high-fat diet. **P*<0.05 vs. NCD+Sham group; ^#^ *P*<0.05 vs. HFD+Sham group; ^&^*P*<0.05 vs. HFD+OA+Vehicle group.
