# Supplementary figures and images for: Salvianolic acid B activates chondrocytes autophagy and reduces chondrocyte apoptosis in obese mice via the KCNQ1OT1/miR-128-3p/SIRT1 signaling pathways
Source: Nutr Metab (Lond). 2022 Aug 3;19:53. doi: 10.1186/s12986-022-00686-0 (PMC9351265; doi:10.1186/s12986-022-00686-0)

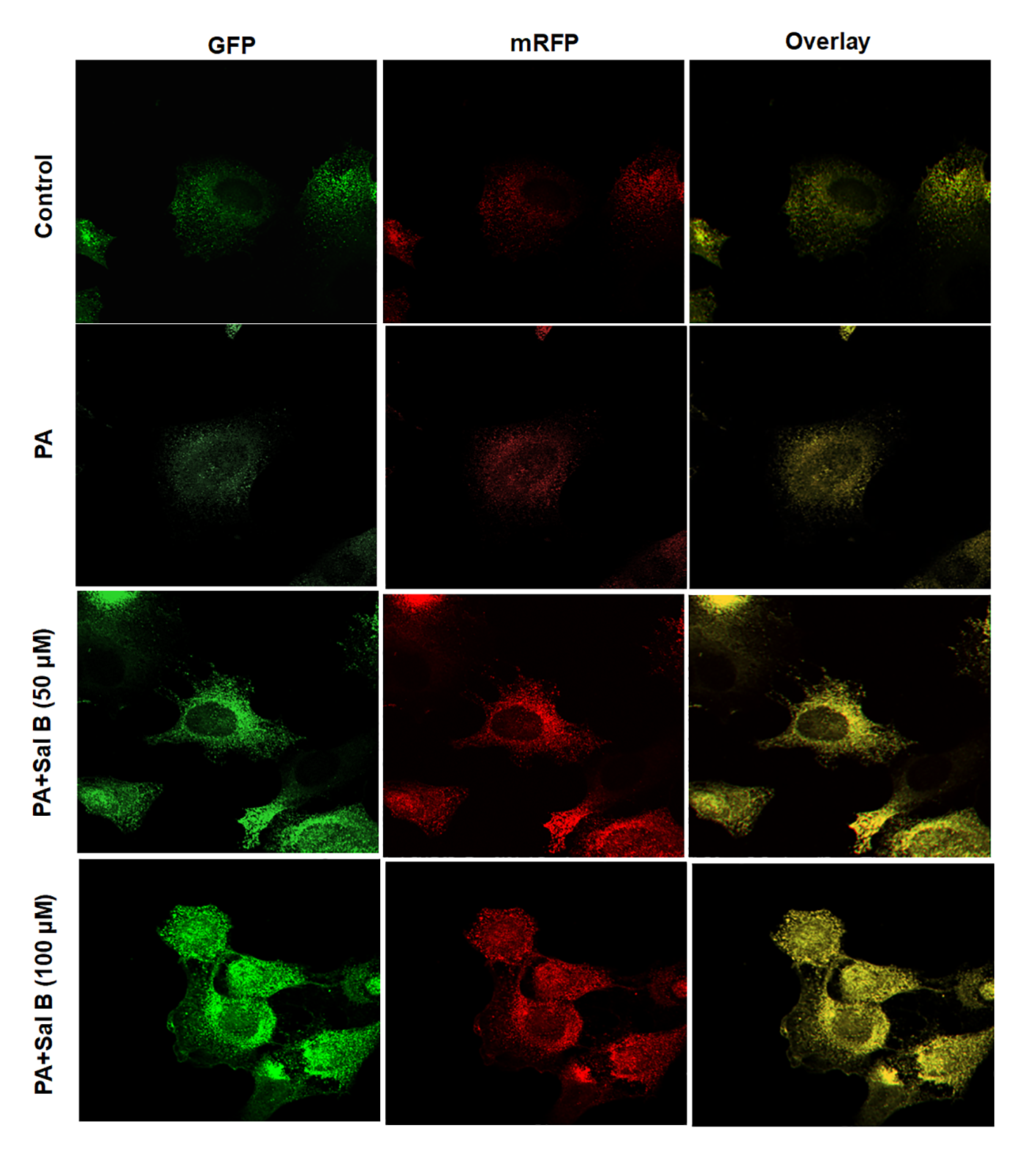

Supplement: Supplementary file 2 — Additional file 2: Fig. S1. Sal B treatment induced autophagy in PA-stimulated ATDC5 cells. Autophagic flux of mRFP-GFP-LC3-transfected ATDC5 cells revealed by laser confocal microscopy. Autophagosomes are labeled by red and green fluorescence (yellow spots), whereas autophagic lysosomes are labeled by red fluorescence (red spots). Magnification: × 1000. [file 12986_2022_686_MOESM2_ESM.tif]

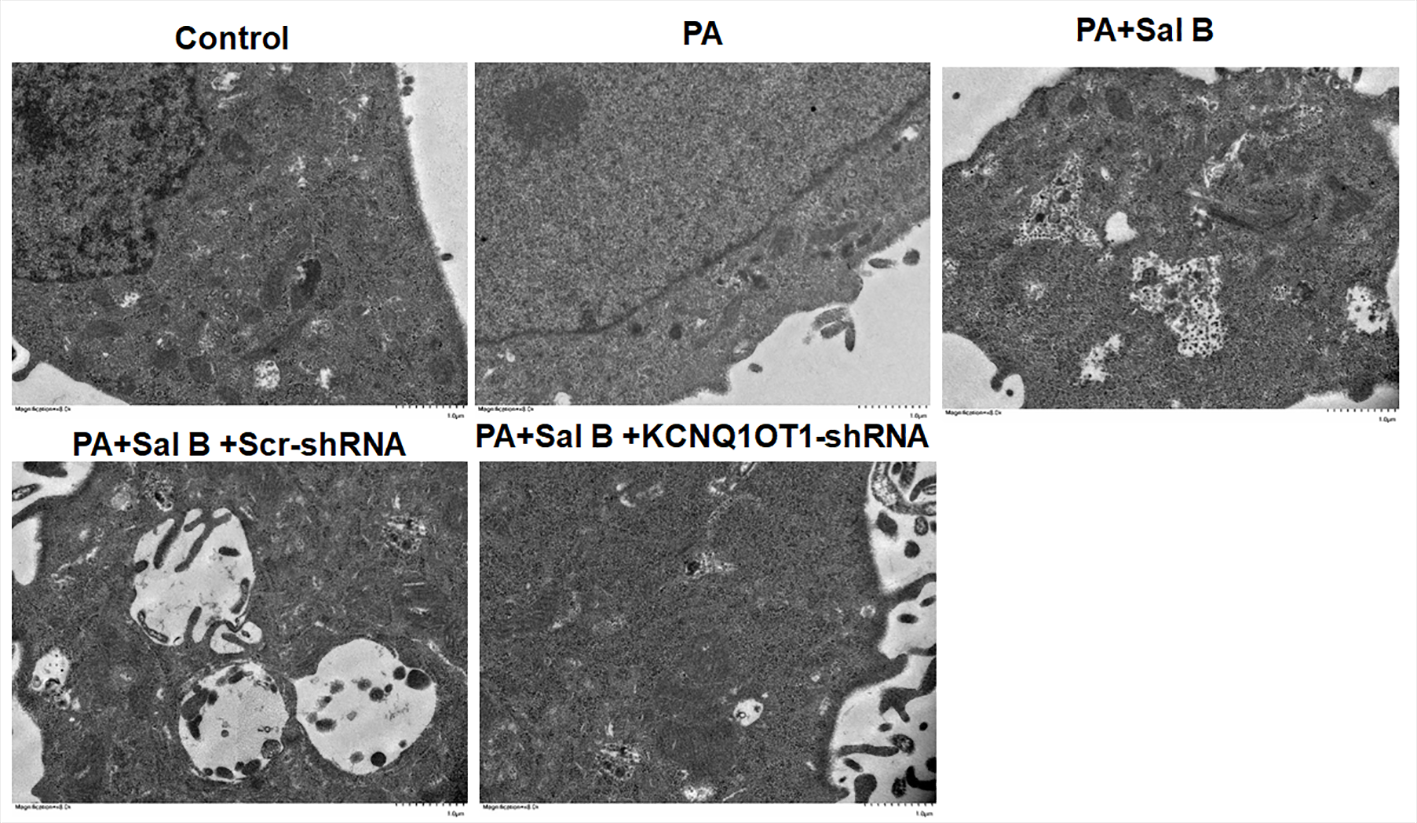

Supplement: Supplementary file 3 — Additional file 3: Fig. S2. Knockdown of KCNQ1OT1 weakened the promoting of Sal B on autophagy in PA-stimulated ATDC5 cells. Transmission electron micrographs of autophagosomes in ATDC5 cells. Magnification: × 8000. [file 12986_2022_686_MOESM3_ESM.tif]
