# Supplementary material for: Salvianolic acid B activates chondrocytes autophagy and reduces chondrocyte apoptosis in obese mice via the KCNQ1OT1/miR-128-3p/SIRT1 signaling pathways
Source: Nutr Metab (Lond). 2022 Aug 3;19:53. doi: 10.1186/s12986-022-00686-0 (PMC9351265; doi:10.1186/s12986-022-00686-0)

**1.** **The details on bioinformatic analysis for the interactions of KCNQ1OT1 and miR-128-3p**

1) RNA hybrid (https://bibiserv.cebitec.uni-bielefeld.de/rnahybrid/)


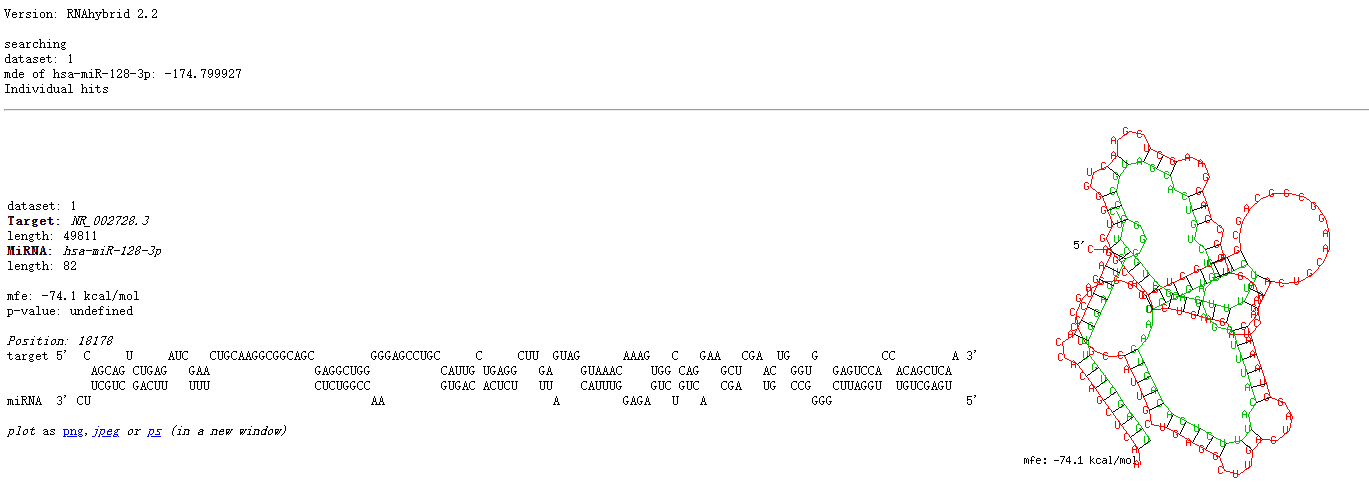


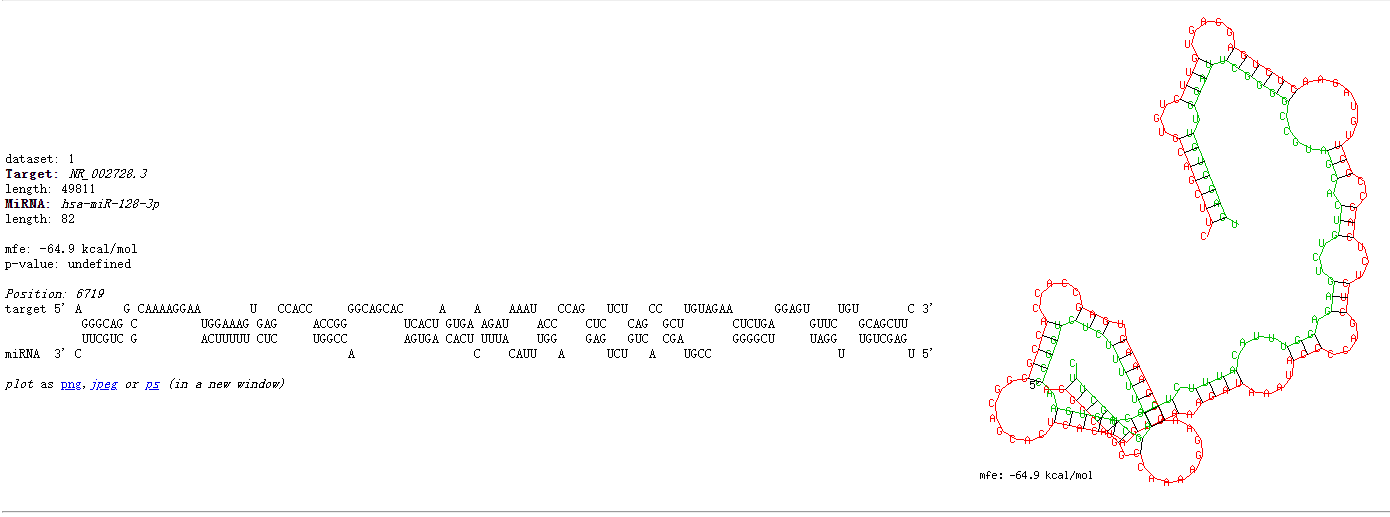


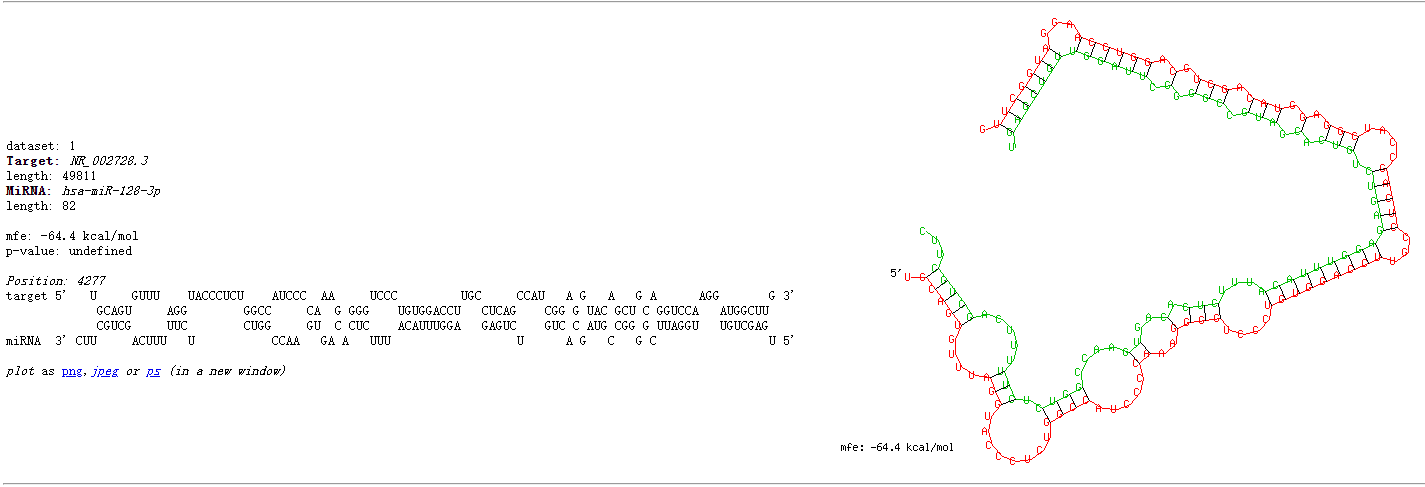


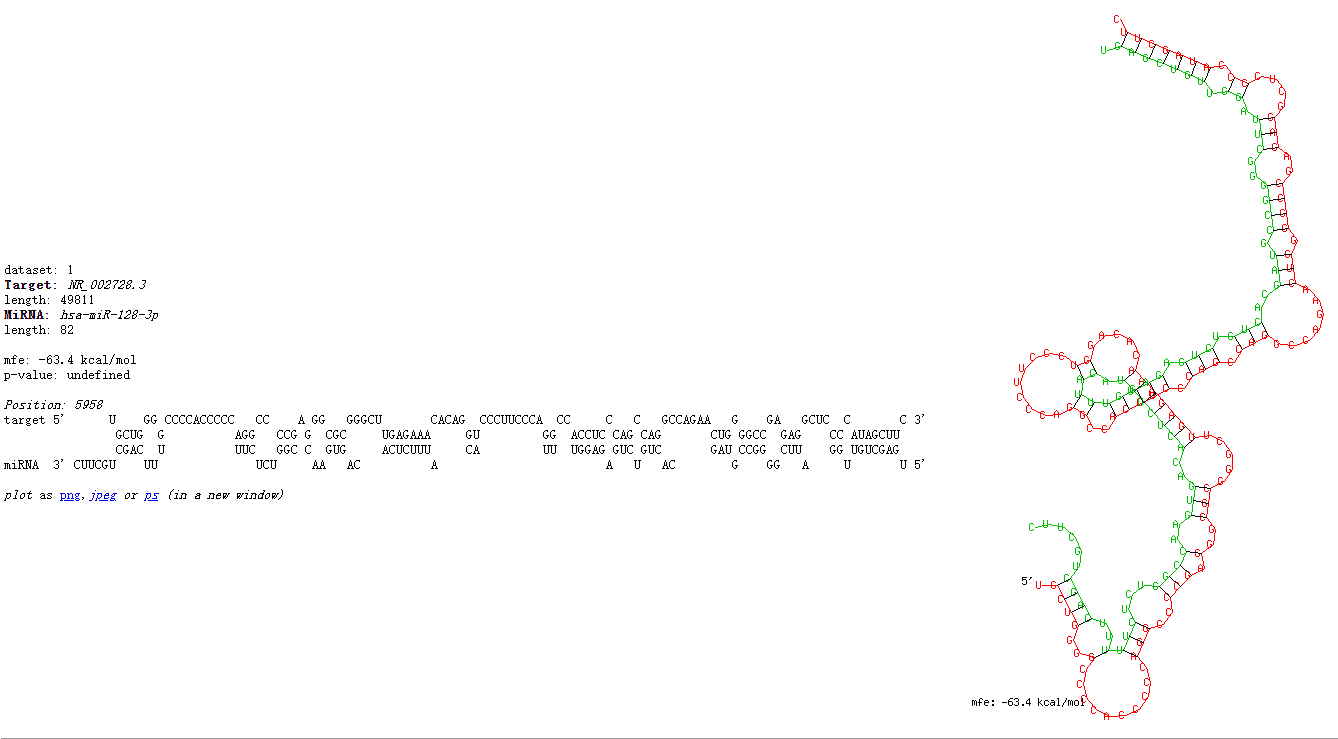


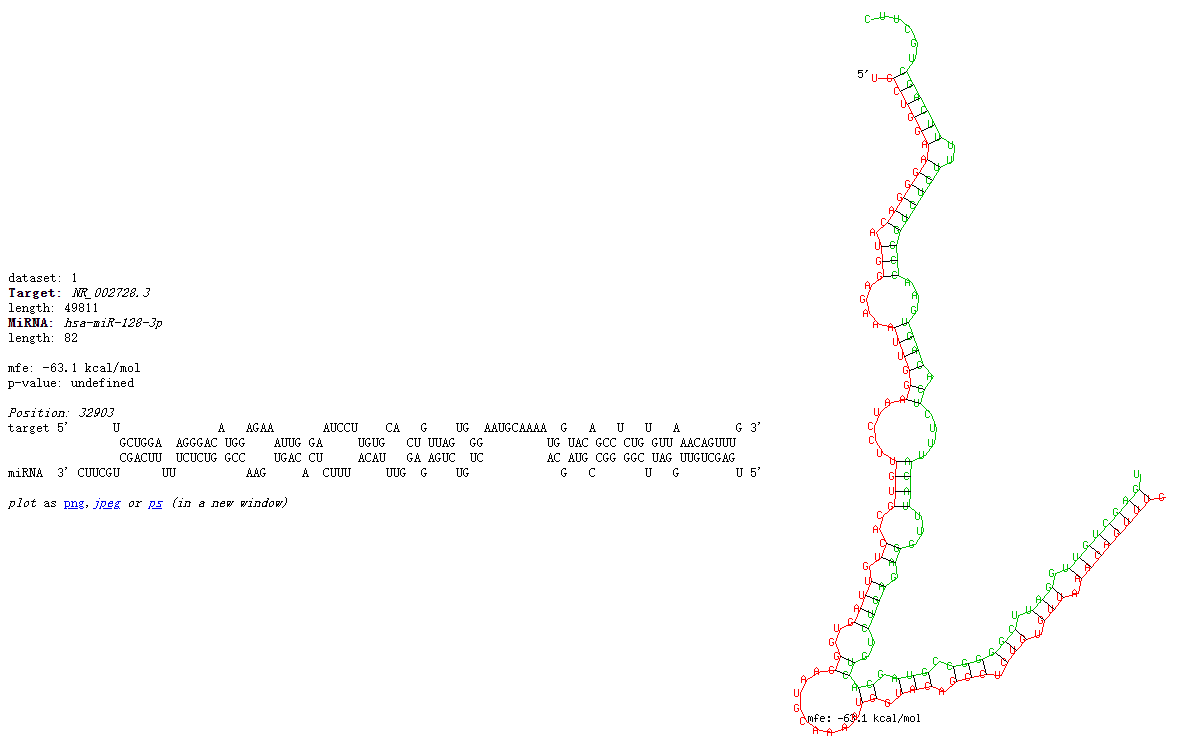


2) StarBase 2.0 (<http://starbase.sysu.edu.cn/>)


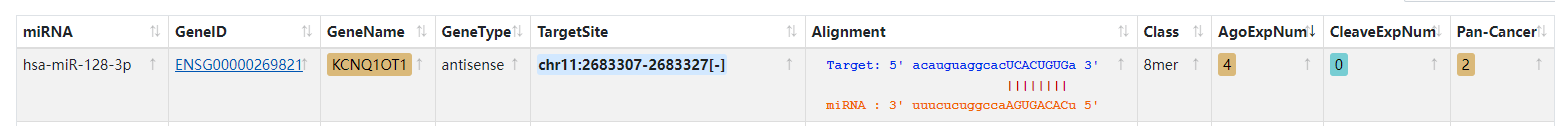


3) DIANA tools (<http://carolina.imis.athena-innovation.gr/diana_tools/web/>)


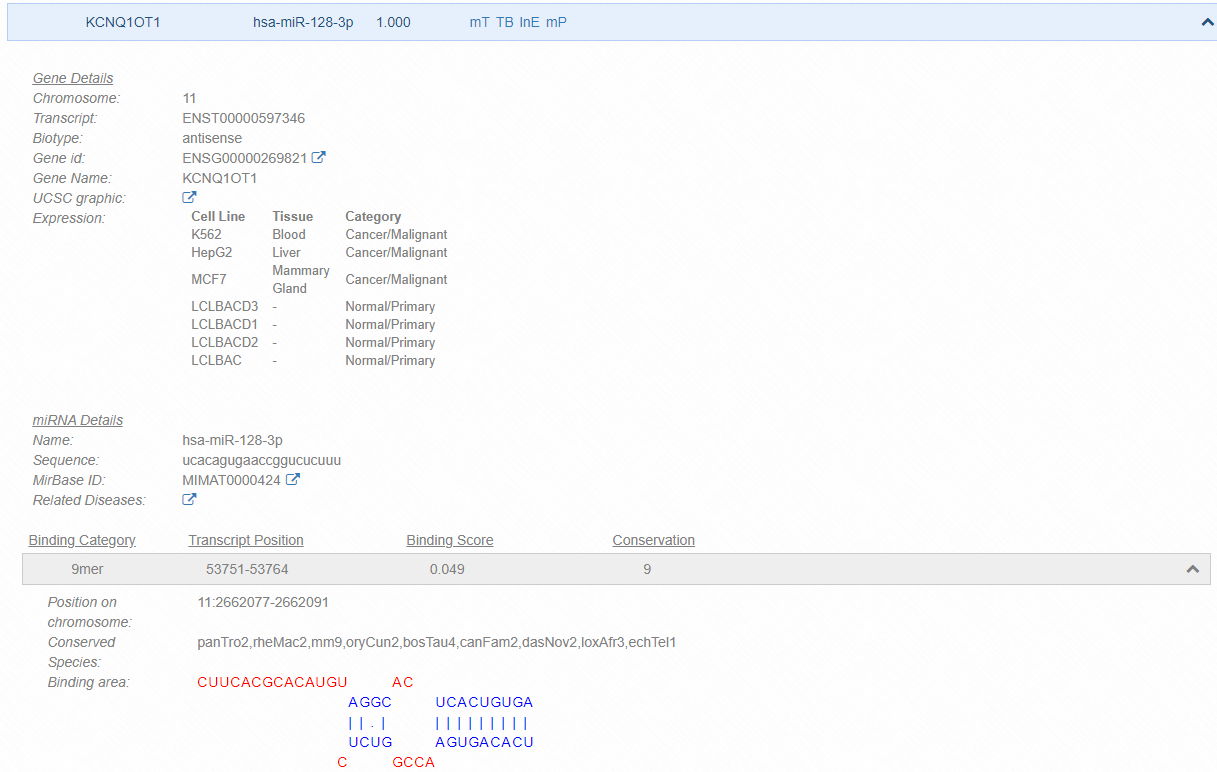


**2.The details on bioinformatic analysis for the interactions of miR-128-3p and SIRT1 mRNA**

1) RNA hybrid (https://bibiserv.cebitec.uni-bielefeld.de/rnahybrid/)


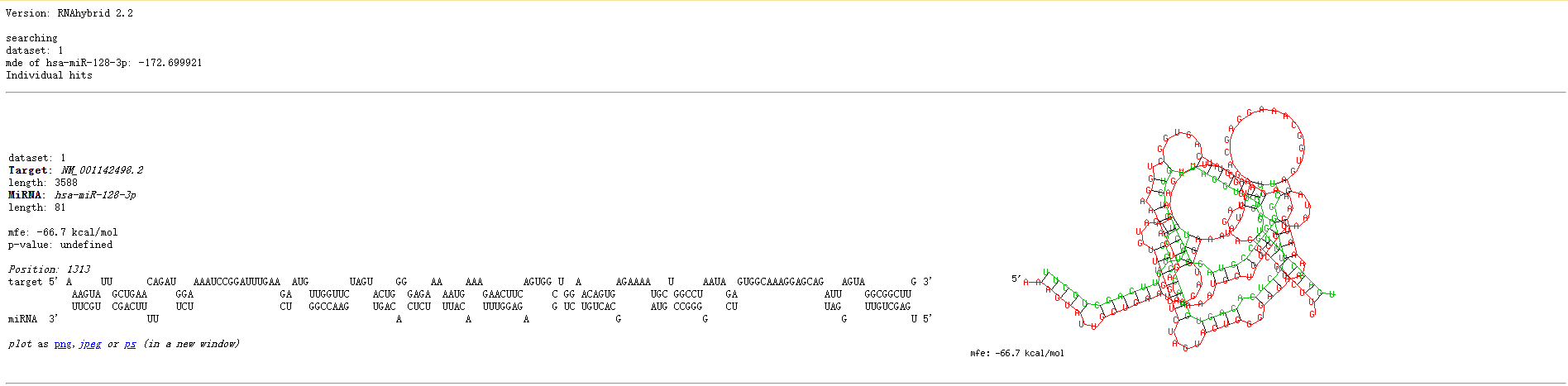


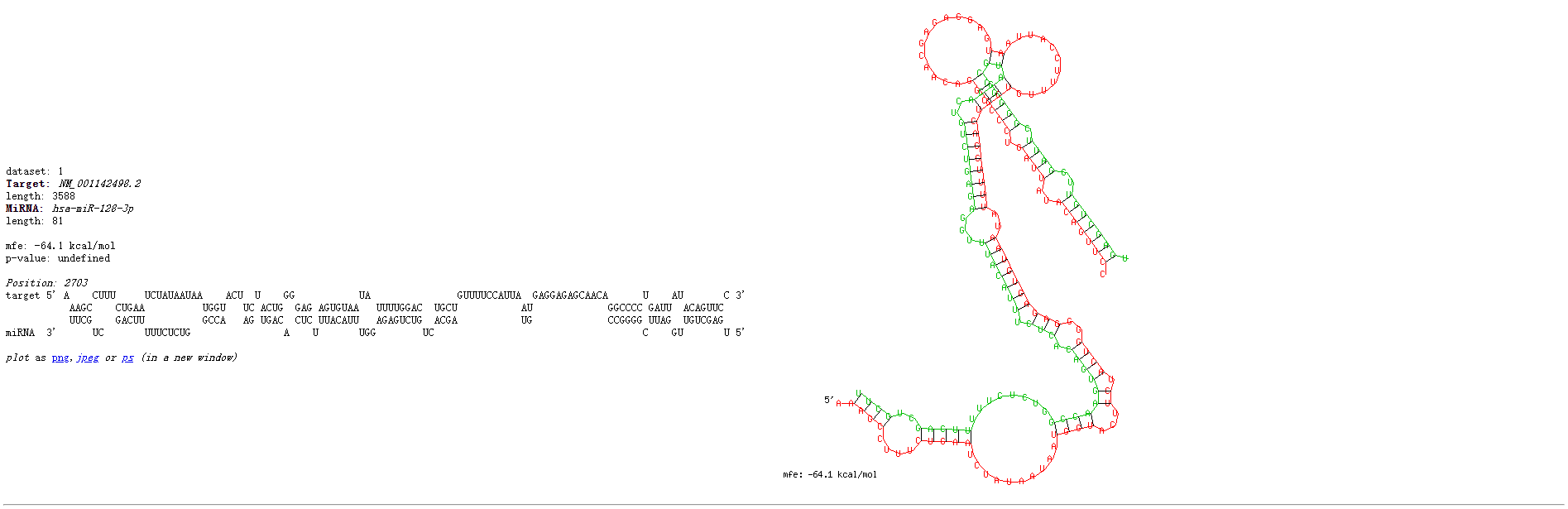


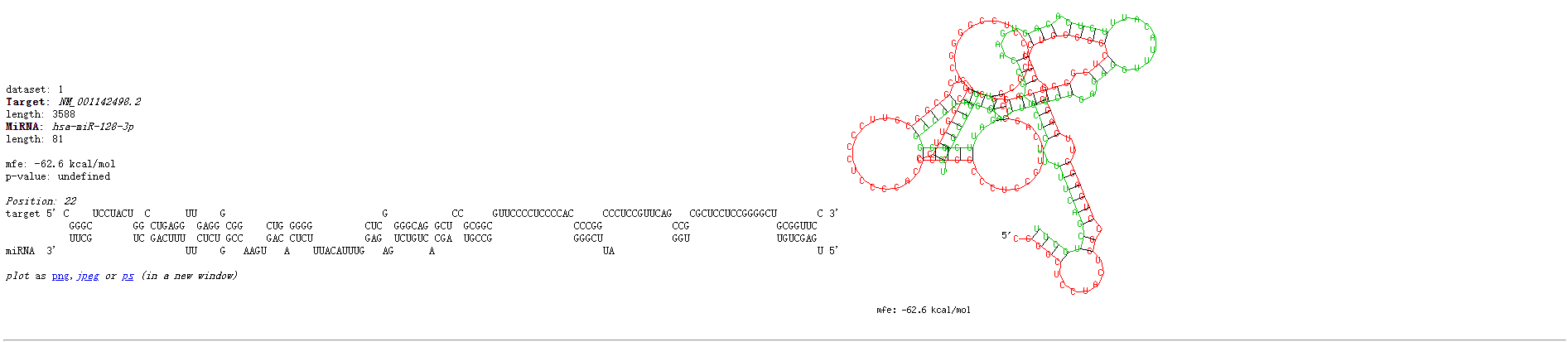


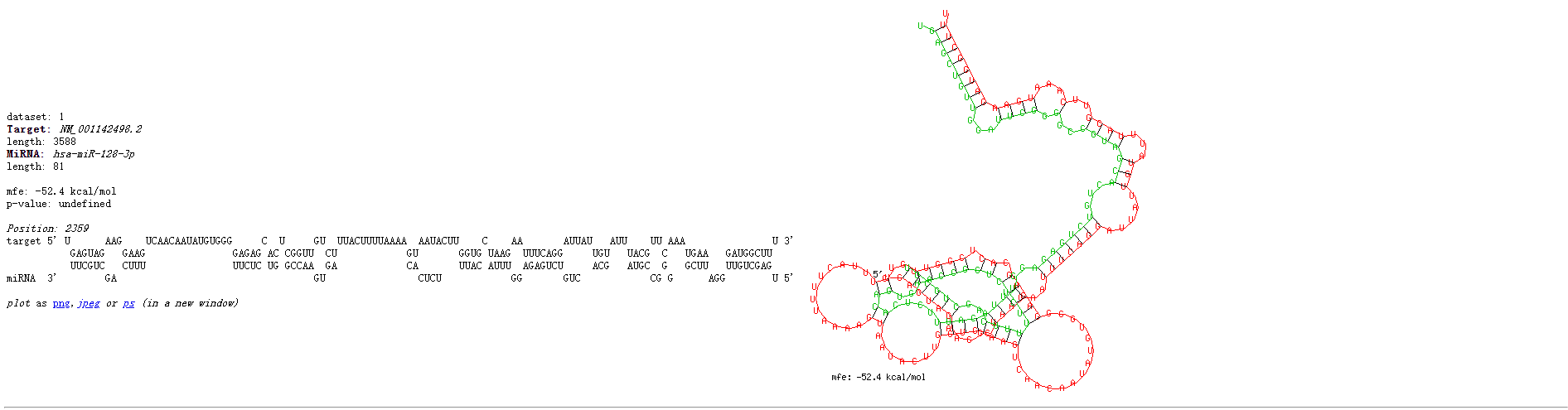


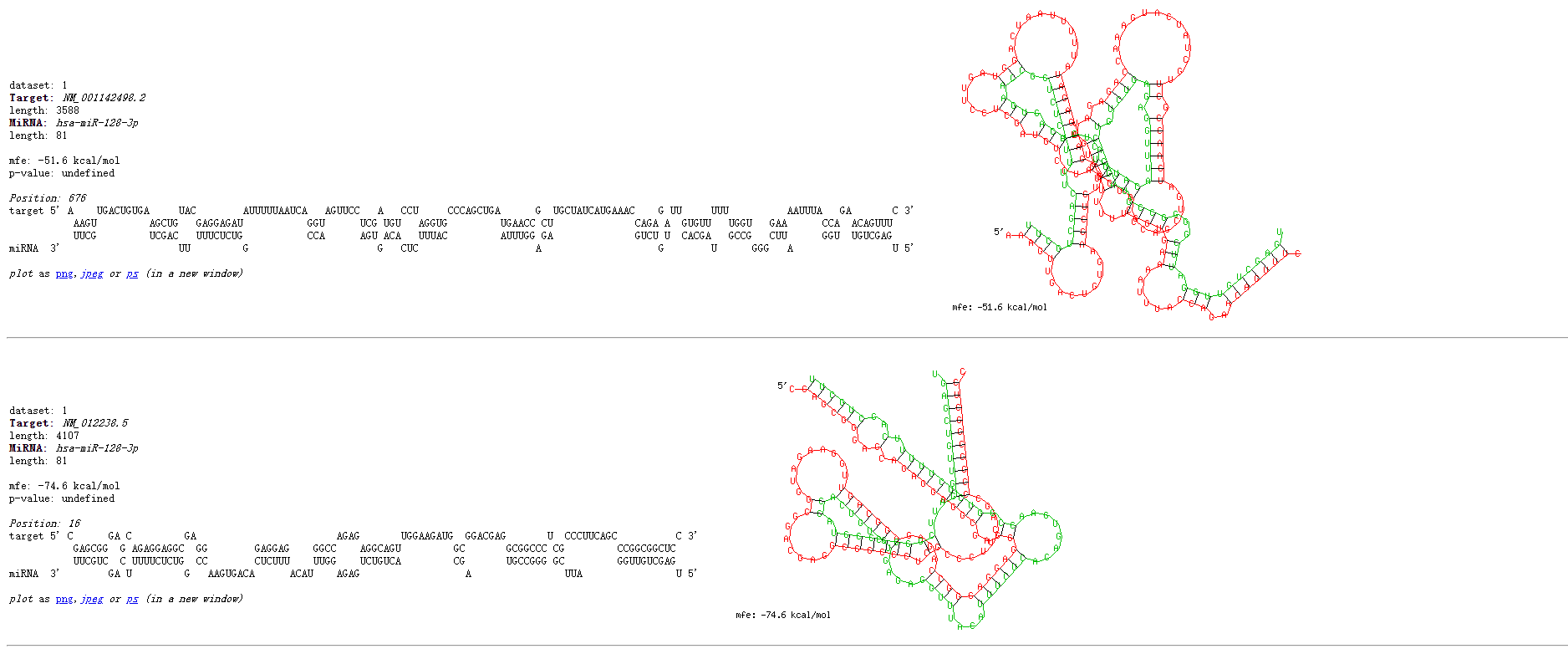


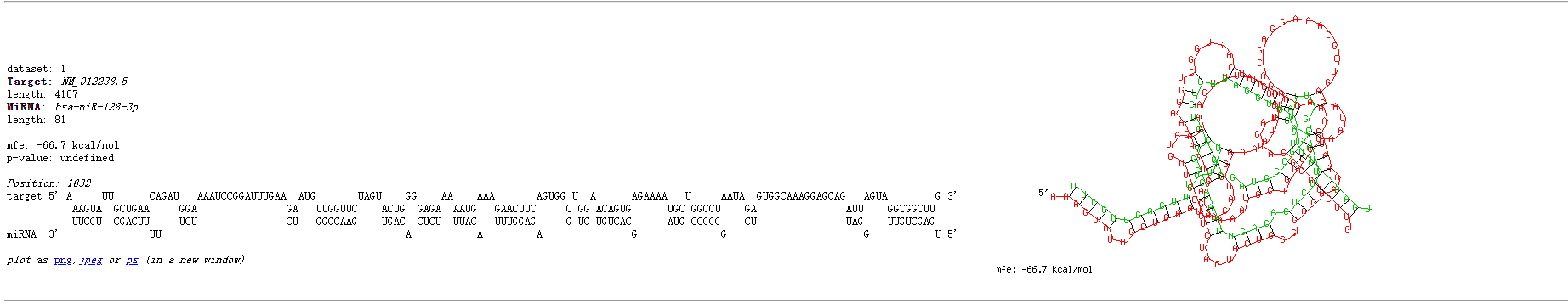


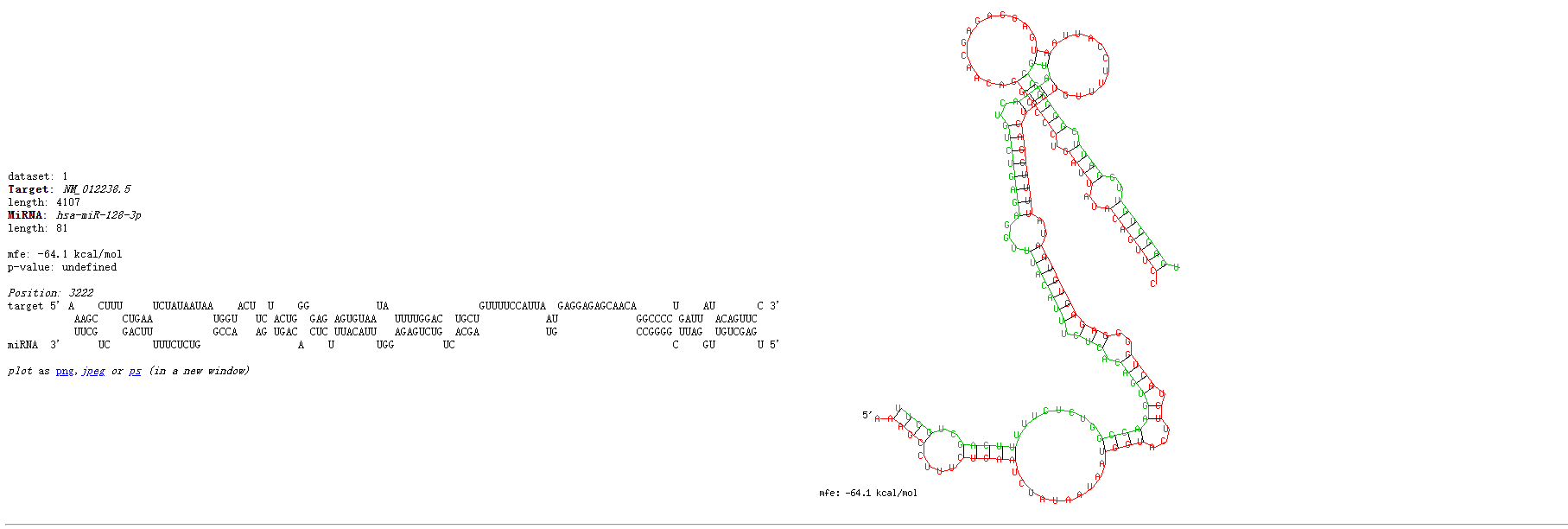


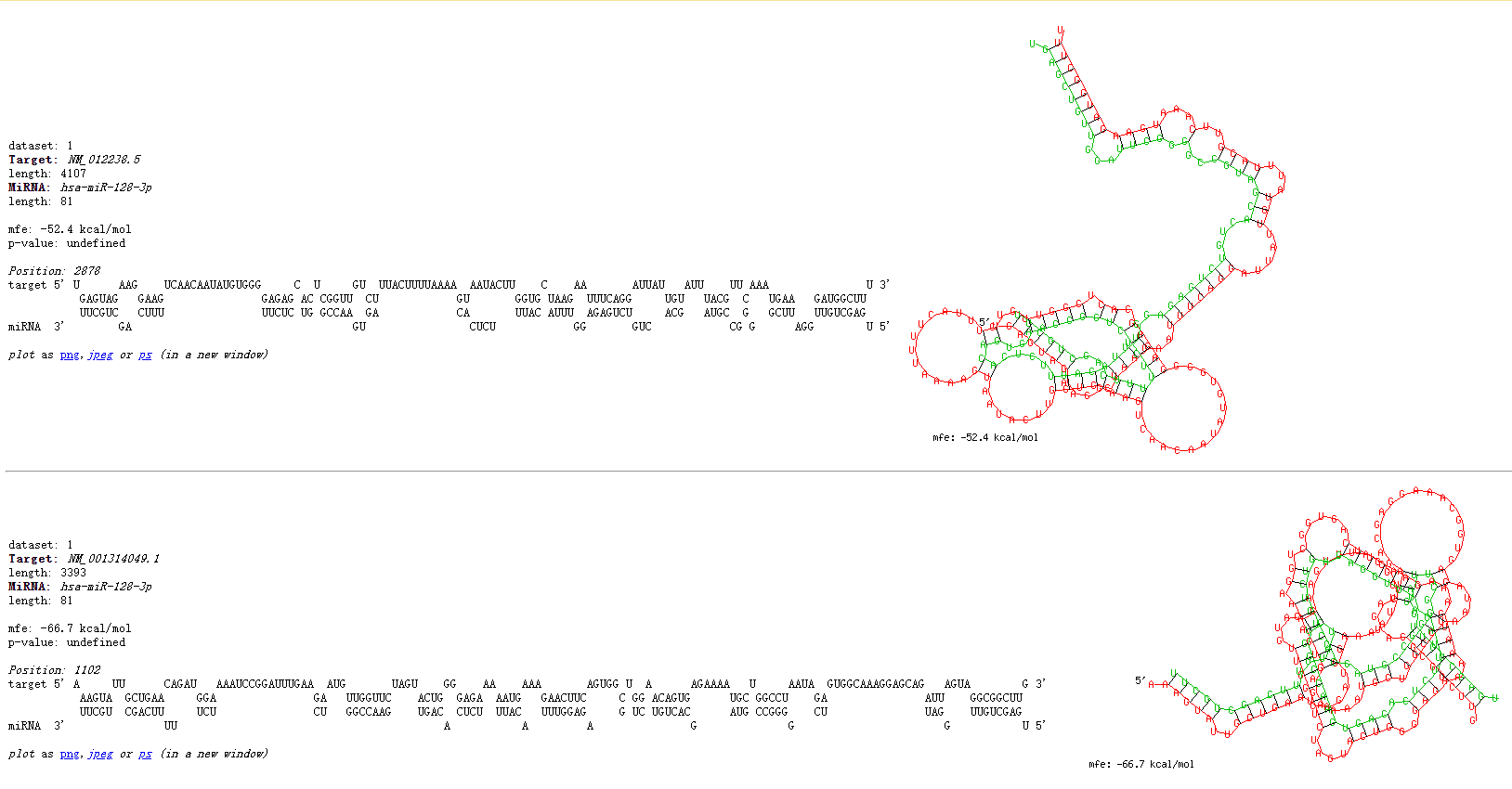

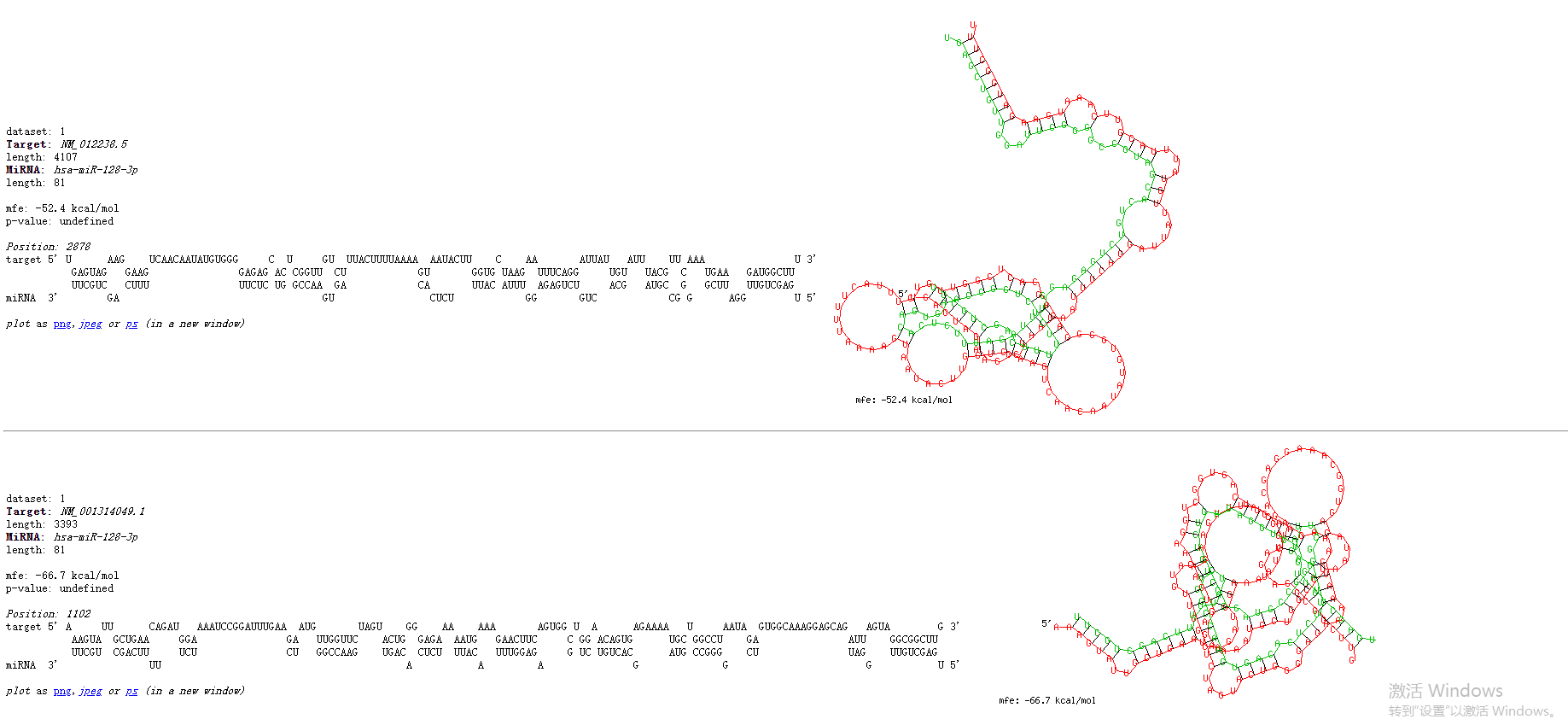


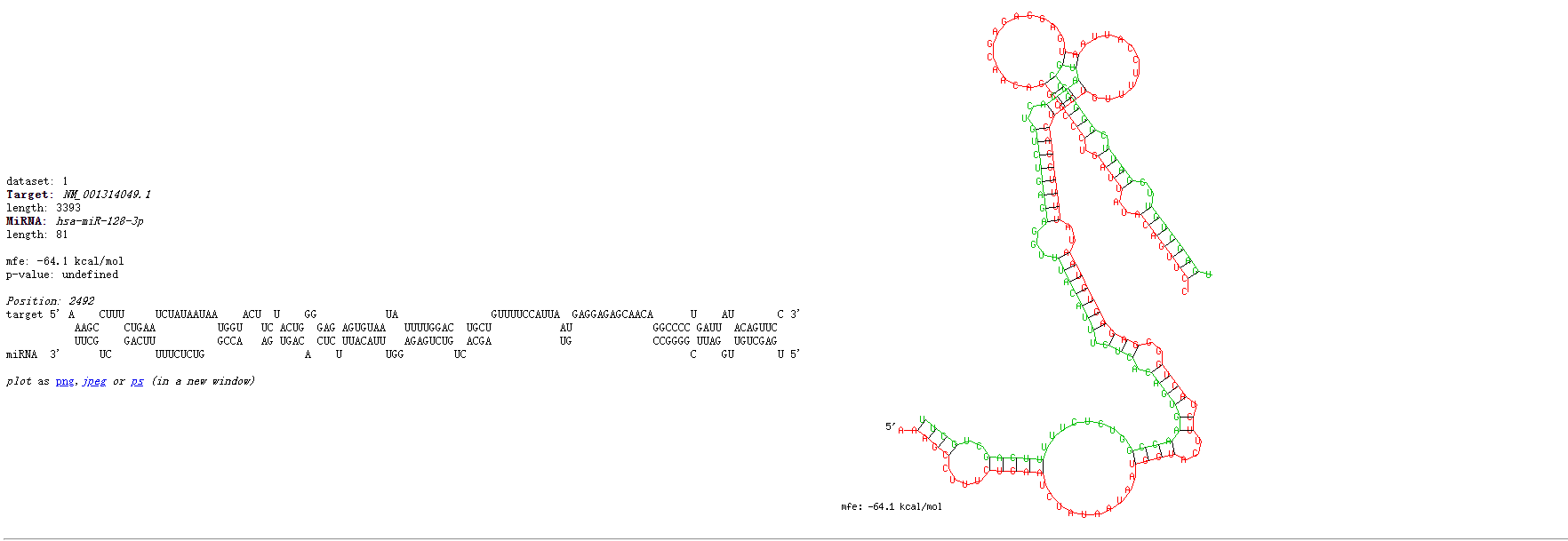


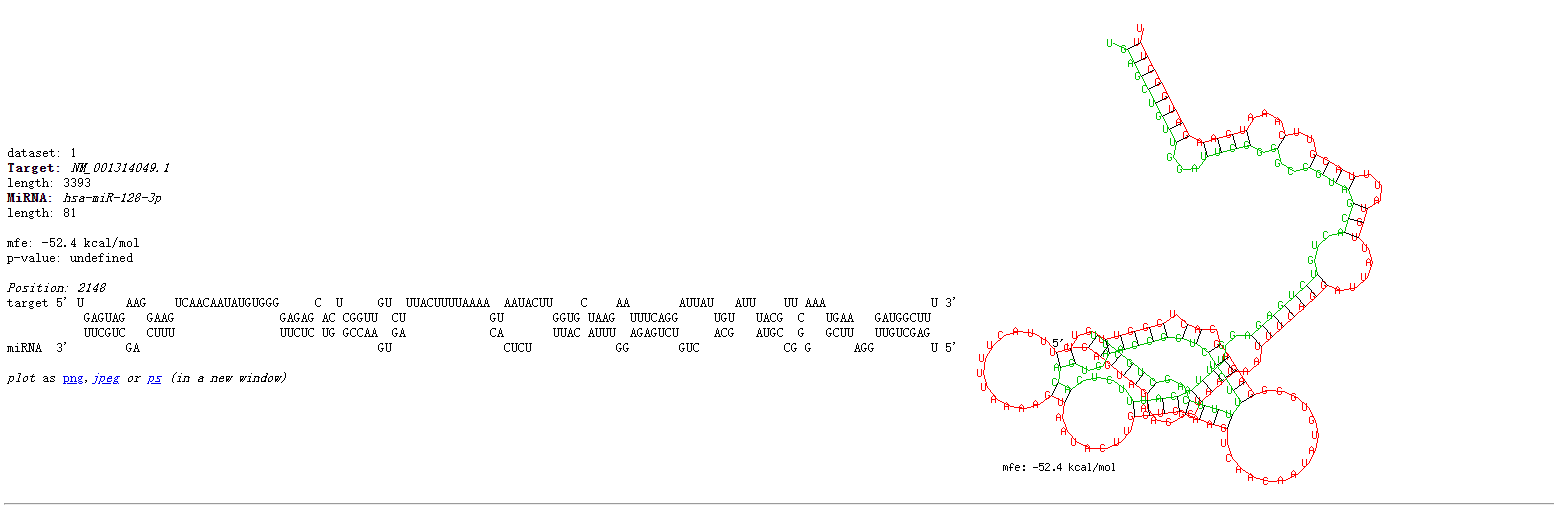


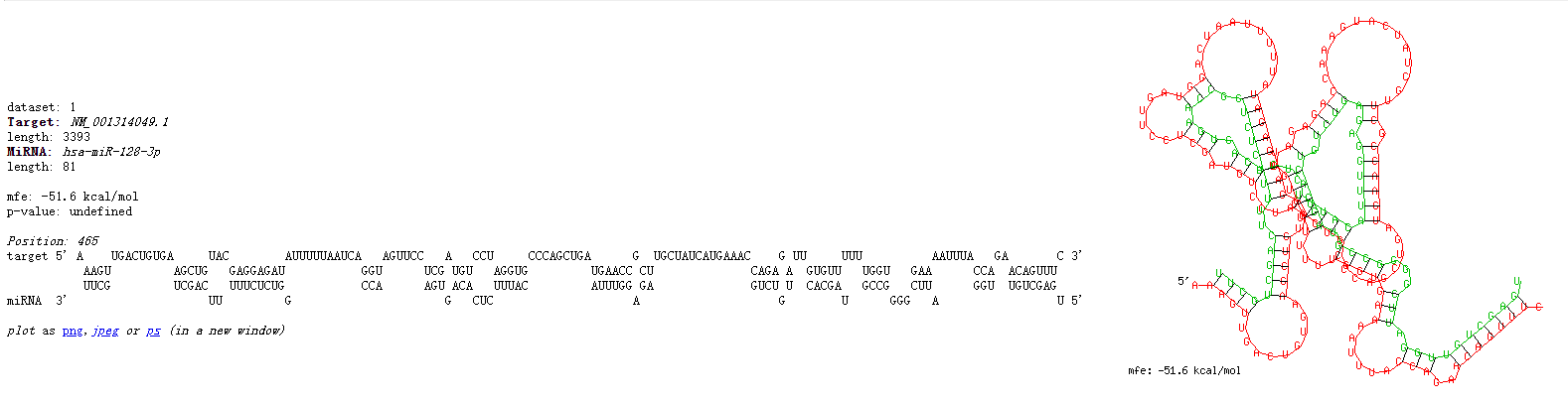


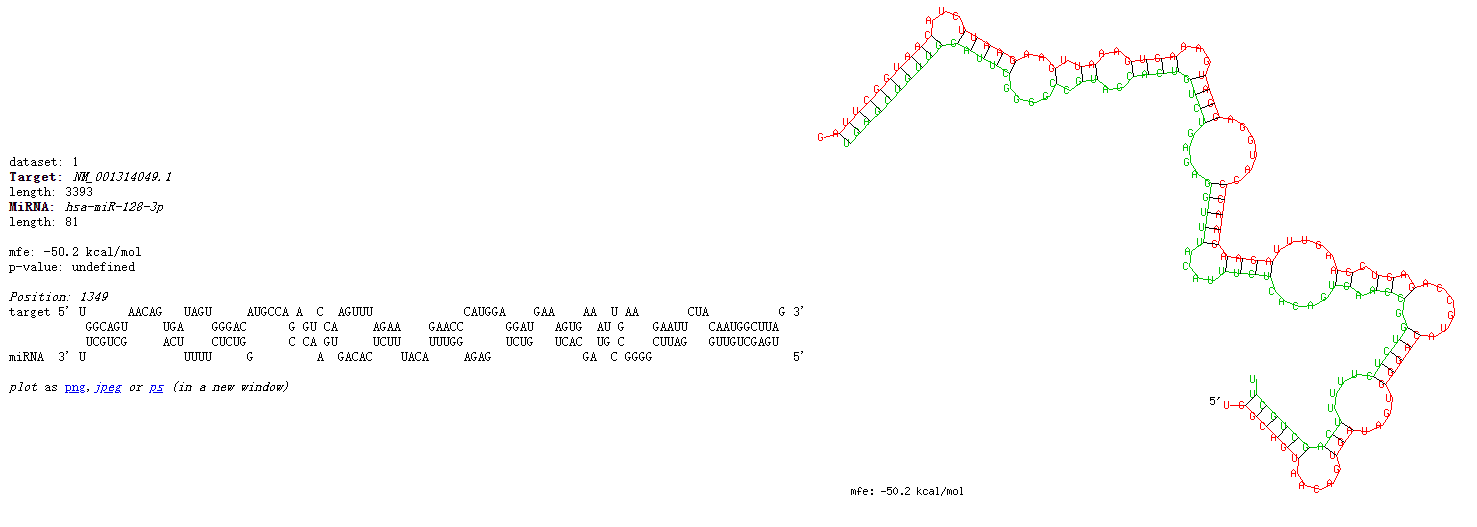


2) TargetScan (<http://www.targetscan.org/vert_71/>)


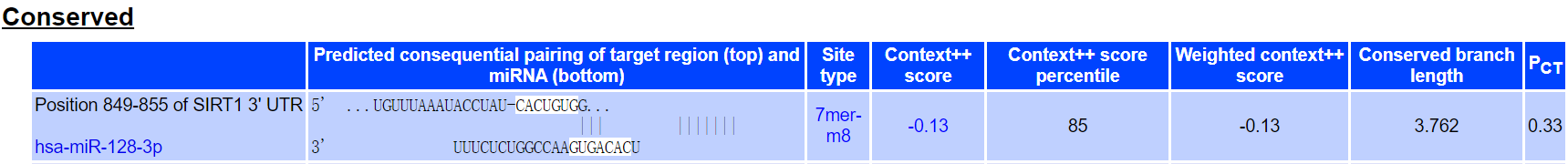


3) miRDB (http://mirdb.org/)


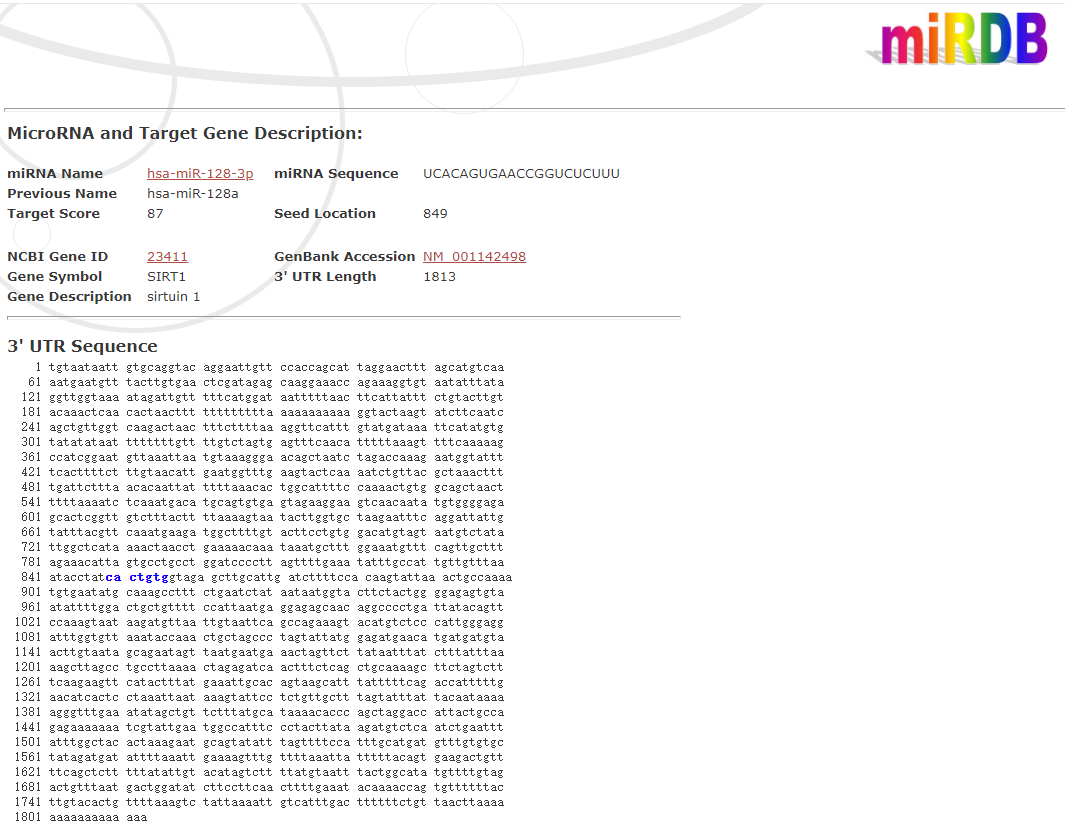

Supplement: Supplementary file 4 — Additional file 4. Prediction for the interactions of KCNQ1OT1 and miR-128-3p by different bioinformatic tools. [file 12986_2022_686_MOESM4_ESM.docx]
